# Chemoselective derivatisation and ultrahigh resolution mass spectrometry for the determination of hydroxyl functional groups within complex bio-oils†

**DOI:** 10.1039/d3ra02779a

**Published:** 2023-06-12

**Authors:** Diana Catalina Palacio Lozano, Hugh E. Jones, Mark P. Barrow, Martin Wills

**Affiliations:** a Department of Chemistry, University of Warwick Coventry CV4 7AL UK diana.palacio-lozano@warwick.ac.uk

## Abstract

Bio-oils are a renewable alternative resource for the production of fine chemicals and fuels. Bio-oils are characterised by a high content of oxygenated compounds with a diverse array of different chemical functionalities. Here, we performed a chemical reaction to transform the hydroxyl group of the various components in a bio-oil prior to characterisation with ultrahigh resolution mass spectrometry (UHRMS). The derivatisations were first evaluated using twenty lignin-representative standards with different structural features. Our results indicate a highly chemoselective transformation of the hydroxyl group despite the presence of other functional groups. Mono- and di-acetate products were observed in acetone–acetic anhydride (acetone–Ac_2_O) mixtures for non-sterically hindered phenols, catechols and benzene diols. Dimethyl sulfoxide–Ac_2_O (DMSO–Ac_2_O) reactions favoured the oxidation of primary and secondary alcohols and the formation of methylthiomethyl (MTM) products of phenols. The derivatisations were then performed in a complex bio-oil sample to gain insights into the hydroxyl group profile of the bio-oil. Our results indicate that the bio-oil before derivatisation is composed of 4500 elemental compositions containing 1–12 oxygen atoms. After the derivatisation in DMSO–Ac_2_O mixtures, the total number of compositions increased approximately five-fold. The reaction was indicative of the variety of hydroxyl group profiles within the sample in particular the presence of phenols that were *ortho* and *para* substituted, non-hindered phenols (about 34%), aromatic alcohols (including benzylic and other non-phenolic alcohols) (25%), and aliphatic alcohols (6.3%) could be inferred. Phenolic compositions are known as coke precursors in catalytic pyrolysis and upgrading processes. Thus, the combination of chemoselective derivatisations in conjunction with UHRMS can be a valuable resource to outline the hydroxyl group profile in elemental chemical compositions in complex mixtures.

## Abstract 2

Mass spectrometry in combination with a targeted derivatisation can be used for profiling molecular compositions containing hydroxyl groups in bio-oils.

## Graphical abstract

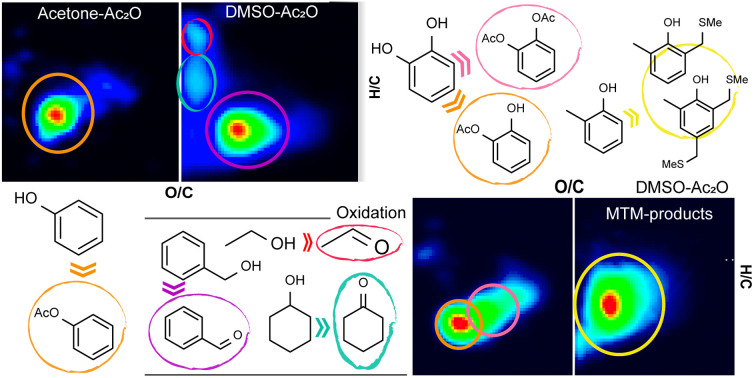

## Introduction

Chemical products such as automotive and industry oil and grease, equipment lubricants, hydraulic oils, food additives, additives for food contact materials, cosmetic and hair-care product ingredients, and additives for pharmaceutical formulations, are mainly produced from highly refined fossil-fuels.^1^ Non-fossil feedstocks, such as those produced by biomass pyrolysis or catalytic depolymerisation of plastic waste, are not only a renewable alternative resource for the decarbonisation of some transport sectors, they are also considered the only readily available source of carbon material to produce chemicals and polymers traditionally produced from refined fossil fuels.^2–4^ Alternative renewable resources can be a key contributor in addressing the challenges of socio-economic growth and climate change mitigation and adaptation.^5^ Bottlenecks in both fundamental knowledge and technological aspects, limit the advances in the chemical processes required for a successful energy and chemistry transition to produce fine chemicals from renewable biological materials.^6^

Biomass is composed of cellulose, hemicellulose, and lignin units.^7,8^ A popular route for the conversion of biomass into liquids (bio-oils) is pyrolysis.^9^ The thermal decomposition occurring in a biomass during a pyrolysis process leads to the formation of thousands of different, mostly oxygen-containing, compositions with a wide variety of monofunctional or multifunctional groups *e.g.* a combination of acids, alcohols, aldehydes, ethers, ethers, furans, phenols, and ketones, among others. In general, bio-oils need further processing to reduce their oxygen content in an effort to improve a bio-oil's quality and miscibility with petroleum fuels.^10^ Previous works have determined that the chemical functionality of model compounds clearly impact the formation of monocyclic and polycyclic hydrocarbons. In general, phenols and cyclic ketones have been shown to convert to a much lower extent under catalytic reactions and to produce mostly coke or other oxygenated compounds.^11–13^ Kim *et al.*,^14^ showed that phenols with saturated side chains (methyl, ethyl and propyl) produced a higher yield of naphthalenes as a consequence of side chain cleavage over a zeolite catalyst and their subsequent oligomerisation, cyclisation, and aromatisation. Due to the negative effect of phenolic compositions, pre-treatments such as methylation of the phenolic hydroxyl groups has been proposed to improve the hydrocarbon production from lignin material.^12^ The structural characterisation of the compositions present in the bio-oil can then facilitate or assist the prediction of some co-processing products *e.g.*, coke and light gases.

Bio-oils are complex mixtures not solely because of the large number of poly-oxygenated compositions, but because each molecule can exist in several isomeric forms, and contain different functional groups,^15^ and therefore exhibit different properties that affect their behaviour under storage, processing, and upgrading. The structural characterisation of such a complex mixture remains a key goal for energy and environmental research. The hydroxyl group is the most abundant functional group in bio-oils^16^ and natural product derivatives^17^ and can be found in carbohydrates, alcohols, carboxylic acids, and phenols. Spectroscopic techniques such as ^31^P NMR (nuclear magnetic resonance) of derivatives can provide valuable quantitative information about the aliphatic phenolic, carboxylic hydroxyl groups, and water content, of bio-oils.^18,19^ The quantitative characterisation by ^31^P NMR is based on the derivatisation with a phosphitylation agent (P-agent) that allows moieties with a hydroxyl group (aliphatic, phenols and carboxylic OH) to react with the P-agent forming an active moiety for ^31^P NMR analysis. Nevertheless, spectroscopic techniques are unsuitable for individual compound analysis within a complex mixture.^16^

A molecular level characterisation of complex mixtures can be achieved with ultrahigh resolution mass spectrometry (UHRMS) techniques. UHRMS techniques are well-known for their ability to resolve thousands of individual molecular compositions with the highest mass accuracy in a single analysis, allowing the assignment of unique elemental compositions.^20,21^ Due to its ultrahigh resolving power and mass accuracy, Fourier transform ion cyclotron resonance mass spectrometers (FTICR MS) are commonly used for the analysis of complex mixtures, allowing access to high-molecular weight compositions in bio-oils and crude oils.^21,22^ The structural identification of the individual compositions in bio-oils is the key goal for a comprehensive chemical composition analysis. Derivatizations as an additional step in sample preparation before UHRMS detection have recently been applied for semi-targeted analysis of functional groups in complex mixtures. Examples include the characterization of thiols in fossil fuels by Michael addition derivatisation,^23^ Ag^+^ complexation for the characterisation of olefin mixtures,^24^ the derivatization of ketone/aldehyde functional groups in weathered petroleum,^25^ the carbonyl group derivatization in an oak pyrolysis bio-oil by the use of 3-chloroaniline,^26^ and the use of esterification reactions to gain insights in reactivity of bio-oil compositions.^15^ Changes in the total abundance of heteroatomic class distributions after derivatisations are a clear indication of the presence of the targeted functional groups in complex mixtures.

Here, UHRMS in combination with derivatisation using acetic anhydride (Ac_2_O) in the presence of different solvents was used to pinpoint those compositions containing a hydroxyl group in a complex bio-oil obtained from lignocellulosic material. We have also included a comprehensive evaluation of the reactions using lignin-derivative standard compounds to gain a greater understanding of the functional group profile changes when the reactions are applied in a complex mixture. The detection of more than 1900 and 18 000 unique compositions observed after reactions in acetone–Ac_2_O and dimethyl sulfoxide (DMSO)–Ac_2_O mixtures, respectively, are a clear indication of the wide variety of structural arrangements of the hydroxyl group within the bio-oil. We have also been able to infer and semi-quantify the presence of primary and secondary alcohols, non-hindered phenols, and aromatic alcohols in bio-oil's elemental compositions.

## Materials and methods

### Chemicals and bio-oil

Twenty lignin-derived model compounds containing a range of hydroxyl groups typically represented in bio-oils were used to evaluate the acetylation reactions.^27^ The standards and their respective identifiers are listed in Table 1. The identifier of each model compound is based on their structural features, *e.g.* Ph for phenols, Ct for catechols and benzene diols, Ox for phenols with oxygenated functional groups, Ol for hydroxyls in primary or secondary positions, and CA for those standards containing a carboxylic acid group. The details of vendors and purities can be found in Table S1.† The acetylation reactions were also performed in a pyrolysis bio-oil obtained from lignocellulosic bio-oil. The bio-oil was purchased from BTG BioLiquids (Enschede, The Netherlands). Briefly, the bio-oil was produced from lignocellulosic biomass in a fast pyrolysis process operating with a short vapor resident time at 450–600 °C, at near atmospheric pressure in the absence of oxygen.

**Table 1:** Structures and their identifier name of the twenty standard molecules

Name	Exact mass	Molecular formula	Identifier
**Small phenols**			
2-Methoxyphenol	124.0524	C_7_H_8_O_2_	Ph-1
2,4-Dimethylphenol	122.0732	C_8_H_10_O	Ph-2
*o*-Cresol	108.0575	C_7_H_8_O	Ph-3
4-Ethylphenol	122.0732	C_8_H_10_O	Ph-4

**Catechols and benzene diols**			
Hydroquinone	110.0368	C_6_H_6_O_2_	Ct-5
1-(2,5-Dihydroxyphenyl)propan-1-one	166.0630	C_9_H_10_O_3_	Ct-6
5-Methylbenzene-1,3-diol	124.0524	C_7_H_8_O_2_	Ct-7
Pyrocatechol	110.0368	C_6_H_6_O_2_	Ct-8

**Phenols with other oxygenated functional groups**			
Ethyl 2-hydroxybenzoate	166.0630	C_9_H_10_O_3_	Ox-9
1-(3-Hydroxy-4-methoxyphenyl)ethan-1-one	166.0630	C_9_H_10_O_3_	Ox-10
3-Ethoxy-4-hydroxybenzaldehyde	166.0630	C_9_H_10_O_3_	Ox-11
4-Hydroxy-3-methoxybenzaldehyde	152.0473	C_8_H_8_O_3_	Ox-12

**Primary and secondary alcohols**			
3-(Hydroxymethyl)phenol	124.0524	C_7_H_8_O_2_	Ol-13
Chroman-4-ol	150.0681	C_9_H_10_O_2_	Ol-14
1-Phenylethan-1-ol	122.0732	C_8_H_10_O	Ol-15
2-Phenylethan-1-ol	122.0732	C_8_H_10_O	Ol-16
3-Cyclohexylpropan-1-ol	142.1358	C_8_H_10_O	Ol-17

**Carboxylic acids**			
2-Hydroxy-3-phenylpropanoic acid	166.0630	C_9_H_10_O_3_	CA-18
2-(4-Hydroxyphenyl)propanoic acid	166.0630	C_9_H_10_O_3_	CA-19
4-Hydroxy-3,5-dimethylbenzoic acid	166.0630	C_9_H_10_O_3_	CA-20

### Acetylation reactions

Each standard was first prepared as a 0.1 mmol in 1 mL (0.1 M) solution in HPLC grade methanol (Sigma-Aldrich, Gillingham, United Kingdom). This sample set was used as a blank to determine the GC retention time of each of the non-derivatised standards. Additionally, a further three sets of each standard were prepared at a concentration of 0.1 mmol in 1 mL (0.1 M) in three different HPLC grade solvents: methanol, dimethyl sulfoxide (DMSO) (Sigma-Aldrich, Gillingham, United Kingdom), and acetone (Avantor, Lutterworth, United Kingdom). Each vial of these three sets was then spiked with 36 μL (39 mg, 0.38 mmol) of acetic anhydride (Ac_2_O 99%, Fischer Scientific, Loughborough, UK). The standards were left to react at room temperature for approximately seven days, after which they were stored at −23 °C until analysis.

Similarly, 79 mg of the bio-oil were weighed into each of 4 scintillation vials, for two sets of reactions. One set of two vials were diluted by the addition of 1 mL of HPLC grade acetone and the other set were diluted by the addition of 1 mL of HPLC grade DMSO. One vial of each set was then spiked with 360 μL (390 mg, 3.8 mmol) of acetic anhydride and were left to react at room temperature. The other pair of vials to which acetic anhydride was not added act as blanks for each reaction. The samples without acetic anhydride (non-derivatised blanks) are named hereafter bio-oil acetone and bio-oil DMSO whereas the derivatised samples are named bio-oil acetone–Ac_2_O and bio-oil DMSO–Ac_2_O.

### Sample preparation

The raw standards and the derivatised standards (a total of 80 samples) were dissolved to a final concentration of 0.1 mg mL^−1^ in methanol before the GC-APCI-TOF MS (gas chromatography-atmospheric pressure chemical ionisation-time of flight mass spectrometry) analysis. The bio-oils before and after derivatisation were prepared at a final concentration of 0.1 mg mL^−1^ in HPLC grade methanol for direct infusion APCI FTICR MS experiments.

### Analytical techniques

High or ultrahigh resolution mass spectrometry techniques were used for the analysis of the samples. High resolution MS coupled to gas chromatography was used for the analysis of standard molecular compositions before and after derivatisation reactions. Direct infusion ultrahigh resolution mass spectrometry was used for the analysis of the more complex bio-oil samples. The experimental parameters are given below.

#### GC-TOF MS

A 7890A GC (Agilent Technologies, Santa Clara, California, USA) was coupled to a GC-APCI II ion source (Bruker Daltonik GmbH, Bremen, Germany) operating in positive-ion mode. The ion source was in turn coupled to a timsTOF Pro (Bruker Daltonik GmbH, Bremen, Germany) for high resolution analysis (45 000 at *m*/*z* 200). The ions were detected in a mass range of 40–1000 Da with a transfer time of 90.0 μs, a collision energy offset of 5.0 eV, and an acquisition rate of 1 spec per sec. The GC TOF MS data obtained for each standard is about 440 MB in size. The chromatographic method was optimised using a standard mixture of even carbon number fatty acid methyl esters (C_4_–C_24_, Sigma Aldrich, Gillingham, United Kingdom). 1 μL of each sample was injected into a 30 m Stabilwax-DA polar phase column (0.25 mmID, 0.25 μm, Thames Restek, UK) with helium (99.9995% purity) as the carrier gas. The GC column is connected to a Rxi guard column (0.25 mmID) using a SilTite μ-union Connector (Thames Restek, UK). The inlet was set at a temperature of 260 °C. The oven temperature was set at an initial temperature of 40 °C for 10 minutes and the temperature was increased at a rate of 5 °C min^−1^ until 255 °C. The oven was then maintained at 255 °C for 10 min. The interface of the GC to the mass spectrometer consists of a heated transfer line that was kept at a temperature of 270 °C. Methanol blanks were run in between experiments to ensure there was no carryover between samples. The standards were ionised in positive-ion mode by a corona discharge needle operating at 2400 nA. Other ionisation parameters include dry gas flow 5.0 L min^−1^, drying gas temperature 220 °C, and nebuliser gas flow rate of 2.0 bar.

#### Ultrahigh resolution mass spectrometry

An APCI II ion source was coupled a 15 T solariX XR FTICR mass spectrometer (Bruker Daltonik GmbH, Bremen, Germany) for the analysis of the bio-oil samples. The ions were detected in broadband magnitude mode with a mass range of *m*/*z* 150–2000. The sample Bio-oil DMSO–Ac_2_O was acquired at 8 MW data set size while the other bio-oil samples were acquired at 4 MW data set size. All data sets were zero-filled once, and Sine-Bell apodisation was applied. The resolving power measured at *m*/*z* 200 for the data at 8 MW was approximately 1 300 000, while the data acquired at 4 MW had a corresponding resolving power of 690 000. The bio-oils were ionised with a corona discharge operating at 1200 nA to reduce in-source fragmentation. Drying gas flows, drying gas temperature, and nebuliser gas flow rate were set at 5.0 L min^−1^, 220 °C, and 2.0 bar, respectively.

### Data processing

#### GC TOF-MS

The mass spectra were processed and analysed with DataAnalysis 5.3 (Bruker Daltonik, GmbH, Bremen, Germany). An average mass spectrum of each chromatogram was calibrated using a homologous series of polyethylene glycol (PEG, column packing). The values representing the overall derivatization yields of each standard were calculated by using a simple area normalization as shown in eqn (1).1
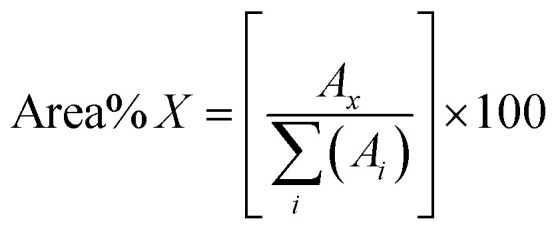
Thus, dividing the area of each peak (*A*_*x*_) in the total ion chromatogram by the total sum of all peaks within that chromatographic profile.

### Data analysis

#### FTICR MS

Sodium trifluoroacetate (Na-TFA, Sigma-Aldrich, Gillingham, United Kingdom) was prepared at 0.1 mg mL^−1^ in methanol and used as an external calibrant. An additional internal calibration was performed in DataAnalysis 5.3 with a calibration list containing abundant homologous alkylated compounds corresponding to protonated molecular compositions containing 2, 5, and 7 oxygen atoms (O_2_[H], O_5_[H] and O_7_[H], respectively). The samples were assigned using Composer 1.5.6 (Sierra Analytics, CA, USA) with molecular assignments corresponding to C_4–100_H_4–1000_O_0–20_N_0–2_S_0–4_ with up to 0.8 ppm error in both protonated (“H”) or molecular ion form. Double bond equivalents (DBE), are calculated according to eqn (2)

2 DBE = *c* − *h*/2 + *n*/2 + 1

where *c*, *h* and *n* are the number of carbon, hydrogen, and nitrogen atoms, respectively. KairosMS and custom R scripts were used for data visualization.^28^ Density diagrams, plotted using VKSim,^29^ were used to visualise the distribution of molecules according to their H/C and O/C-values. The total number of compositions are reported including isotopic compositions unless otherwise specified. The intensity-weighted mean of the oxygenated heteroatomic classes, O_o_, is calculated using eqn (3)^30^3
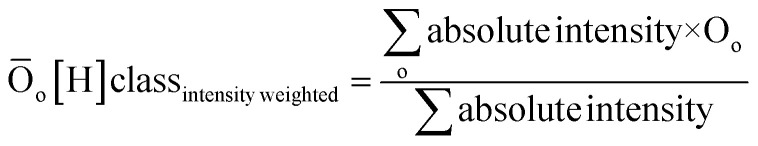


## Results and discussion

### GC-TOF MS of lignin-representative compounds

Bio-oils are distinguished from fossil-fuels by their characteristic content of highly oxygenated chemical compounds. The chemical profile of bio-oils can include aromatic and aliphatic hydrocarbons, phenolic derivatives, carboxylic acids, esters, ethers, and chemicals containing an array of different functional groups. Identifying the specificity of the conversion of the hydroxyl moiety amidst an array of more reactive functional groups then needs to be evaluated. In this section, acetic anhydride in the presence of acetone, methanol and DMSO was evaluated to transform the hydroxyl group in lignin-representative model compounds.

**Fig. 1:**
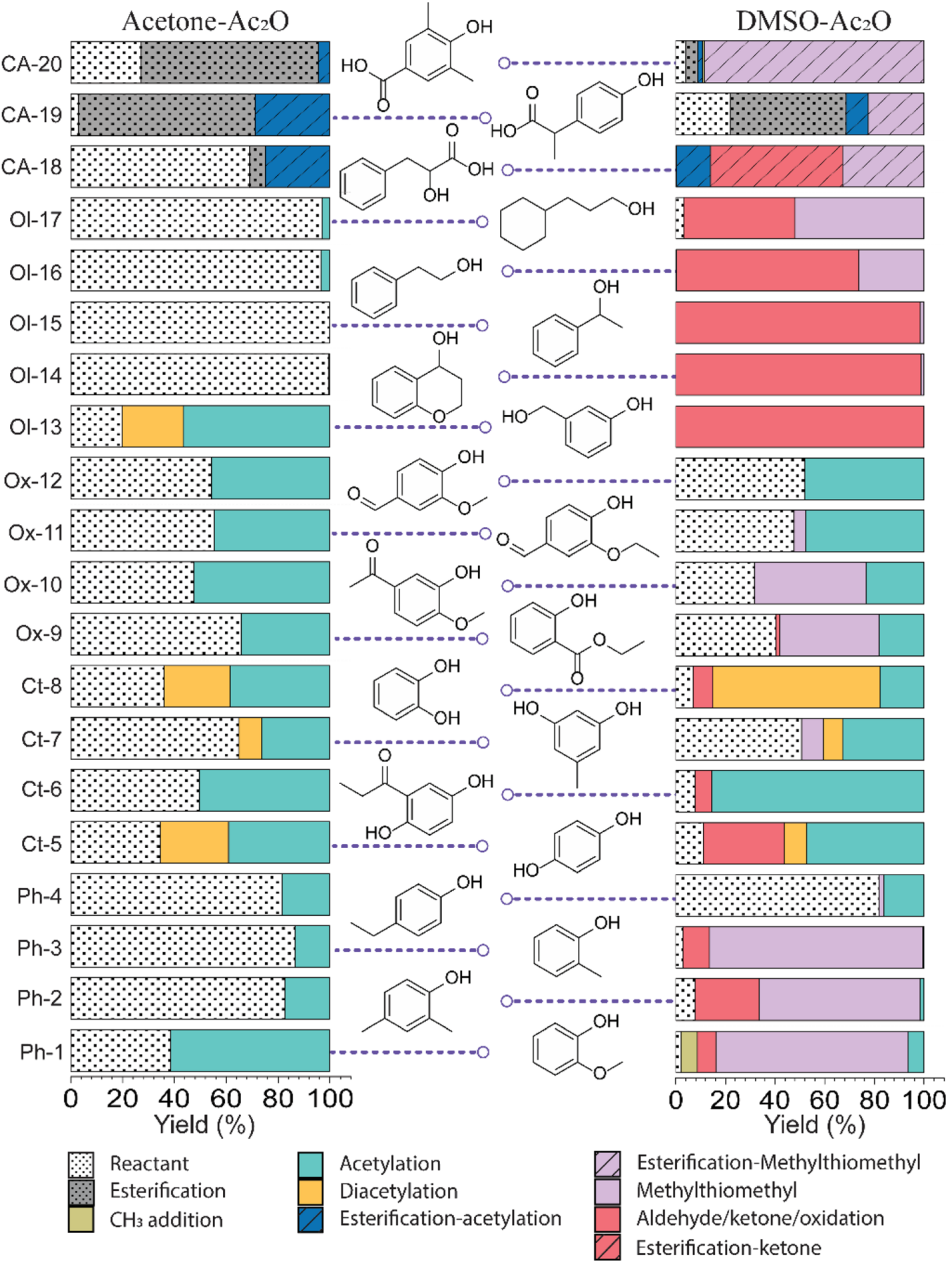
Reaction yields observed on lignin-representative standard molecules in mixtures of acetone–Ac_2_O and DMSO–Ac_2_O.

Standards before and after reactions in acetic anhydride were characterised by GC-TOF MS. GC-TOF MS allows the identification of the raw standards and reaction products based on their retention time, and additionally generates smaller data set size files in comparison with GC-FTICR MS.^28^ The chromatograms and relevant extracted ion chromatograms can be found in Fig. S1–S20 in the ESI.† The retention time of the raw standards was used to identify the respective reactant and products of the reactions with acetic anhydride, and the yields were then calculated using eqn (1). A list of the retention times and their relative abundances can be found in the ESI† (“Retention time data.xlsx”). A summary of the products of the reactions are shown in Fig. 1 and an extended version of the reaction products can be found in Fig. S21 and S22.†

The standards analysed in this work correspond to several types of phenols, aromatic/cyclohexane alcohols, phenols with other functional groups (ester, ethers, ketone, aldehydes and carboxylic acids), and compounds that contain up to two hydroxyl groups. As can be seen in Fig. S1–S20,† the raw standards were shown to be ionisable by APCI. Most of the standards were detected as [M + H]^+^ ions. It is noticeable however, that standards with a hydroxyl group in a primary or secondary position, *e.g.*, Ol-13, Ol-14, Ol-15, Ol-16, and Ol-17, were detected as either an odd-electron ion [M]^+^˙ or a pseudo-molecular [M − H]^+^ ion.

### Derivatization of lignin-representative model compounds

Fig. 1 summarises the results of the derivatisation studies in acetone and in DMSO; full details including proposed reaction mechanisms can be found in Section 1.3 of the ESI.†

#### MeOH–Ac_2_O mixtures

Acetylation reactions were effectively not-observed when the standards reacted in a mixture of MeOH–Ac_2_O (see Fig. S21†), presumably due to a rapid competing reaction of methanol with the acetic anhydride. The selective acetylation of the hydroxyl group is therefore affected if hydroxyl group-containing molecules such as MeOH are present in excess. A similar effect has been reported for the transformation of hydroxylic groups of biological samples which are rich in water.^31^ It is expected then that acetylation reactions in bio-oils, which typically contain between 15 and 30% of water, would give unsatisfactory reaction yields in MeOH–Ac_2_O mixtures. It is however interesting to note that a side-reaction corresponding to the esterification of the carboxylic acid group of CA-18, CA-19 and CA-20 was observed with a high yield in MeOH–Ac_2_O. The highest esterification yield was observed in CA-19 (98%) followed by CA-18 (48%) and CA-20 (27%). Although the reaction of MeOH with Ac_2_O will generate acetic acid as a product which can then catalyse the esterification of carboxylic acids in alcohols in the absence of acid catalysts; self-esterifications have been previously reported *i.e.*, maleic acid in methanol and 2-hydroxyisobutyric acid in methanol.^32,33^ Sangwon Kim *et al.*,^33^ suggested that molecular 2-hydroxyisobutyric acid efficiently catalysed its own esterification while methanol served as both reactant and solvent. Direct infusion experiments of the standards containing a carboxylic acid (data not shown) prepared at 0.1 mg mL^−1^ in only DMSO, or only acetone, indicated that the esterification reactions also occur in the absence of MeOH, suggesting that CA-18, CA-19 and CA-20 are self-esterified in the solution.

#### Acetone–Ac_2_O mixtures

A variety of acetate ester products with various yields were obtained when the reactions were performed in acetone–Ac_2_O. As can be seen in Fig. 1, Ol-14, Ol-15 Ol-16 and Ol-17 were to a great extent non-reactive under the experimental conditions (yield < 3%), whereas Ol-13 was shown to be highly reactive (about 80% yield). Ol-14, Ol-15 Ol-16 and Ol-17 correspond to compositions containing a primary or secondary alcohol group without a phenol group. In contrast, Ol-13 corresponds to a hydroxybenzyl alcohol that is phenol substituted at position C-3 by a hydroxymethyl group. Our results indicate that the major product corresponds to 3-(hydroxymethyl)phenyl acetate (43%), followed by 23% for the di-acetate product (3-acetoxybenzyl acetate), and only 8% of acetylation in the hydroxymethyl group to form 3-hydroxybenzyl acetate (see the ESI†). Therefore, the presence of a phenol group seems to be responsible for the higher yield of acetylation in Ol-13. The acetate formation was hindered by the presence of methyl groups in the aromatic ring *e.g.*, Ph-2, Ph-3, Ph-4, Ct-7, and CA-20 (with yields <20%) whereas a yield of 44–62% was obtained for Ox-10, Ox-11, Ox-12 and Ph-1, all of which are phenols with ether groups. In contrast to ether groups, the ester group in Ox-9 seems to hinder the acetylation reaction. Similar product yields of the mono and diacetate product were observed in hydroquinone and catechol (Ct-5 and Ct-8, respectively), whereas the presence of a ketone group in Ct-6 seems to inhibit the formation of a diacetate product. In summary, an acceptable reaction yield of the phenolic standards in acetone–Ac_2_O mixtures was observed except in cases of sterically hindered phenols. Additionally, primary, and secondary alcohols are essentially non-reactive when the acetylation reaction is carried out in acetone. This provides a valuable method for differentiation of hydroxyl group types in complex mixtures.

#### DMSO–Ac_2_O mixtures

**Scheme 1:**
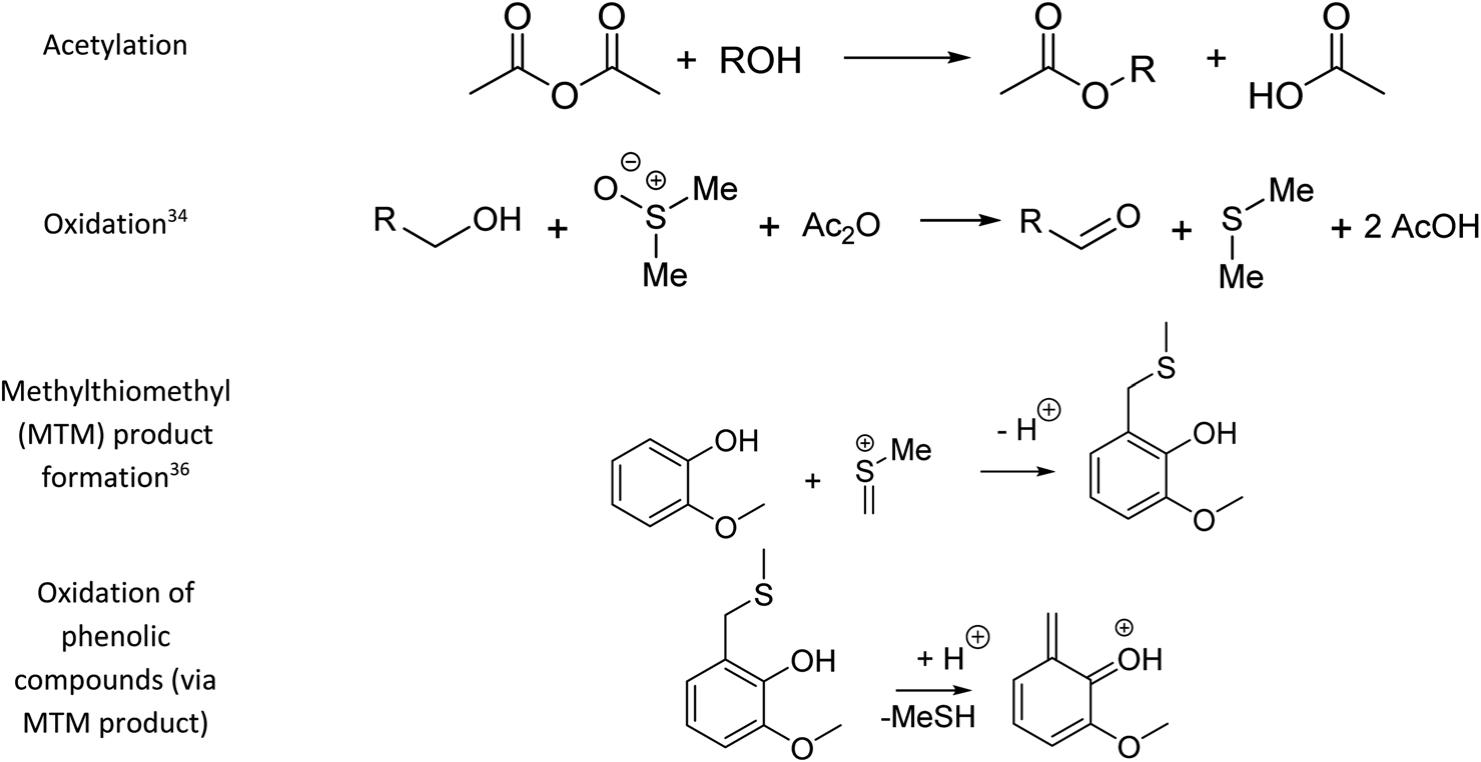
Main reactions observed in the derivatisation of lignin-representative molecules.

The mixture of dimethyl sulfoxide and acetic anhydride, also known as Albright–Goldman reagent, is typically used for the oxidation of primary and secondary alcohols to aldehydes and ketones, respectively.^34^ The reaction of sterically hindered alcohols (*e.g.* indole alkaloids, carbohydrates, and steroids) can lead to a varied yield of *O*-methylthiomethyl (*O*-MTM) derivatives,^34,35^ whereas phenols can be methylthiomethylated in DMSO–Ac_2_O in an available *ortho*-position (MTM attachment).^36,37^ In contrast with acetone–Ac_2_O and MeOH–Ac_2_O, some standards, notably the catechols and benzene diols, prepared in DMSO presented an evident colour change after the reaction was performed (see Fig. S1–S20†). For instance, Ct-5, Ct-6 and Ct-8 turned dark brown in DMSO–Ac_2_O, while others such as Ol-14, Ol-15 and Ol-16 remained colourless after the reaction and others such as Ph-1, Ph-2 and Ph-3, turned light-yellow. This may be the result of catechol and phenol oxidation (see discussion below) followed by a polymerisation to a small amount of a highly coloured product in these cases. Therefore, it is expected that a very distinctive reactivity, combining esterification, oxidation and methylthiomethylation, of the standards exists in DMSO–Ac_2_O mixtures. The results can be found in Fig. 1 and S22.† A summary of the main reaction mechanisms observed can be found in Scheme 1.

Ol-14, Ol-15, Ol-16, and Ol-17, that were shown to be non-reactive with Ac_2_O in acetone and MeOH mixtures, gave products in excellent yield in DMSO–Ac_2_O mixtures (yields > 97%). The hydroxyl groups in Ol-14, Ol-15 and to a lesser extent CA-18 (the former, a composition with secondary alcohol and a carboxylic acid functional group) were oxidised to form a ketone under the experimental conditions, whereas Ol-13 and Ol-16 presented a combined reaction of acetylation and oxidation as major product. In Ol-13 for instance, an acetate ester of the phenol plus aldehyde formation from the primary alcohol were observed with a yield of 84%. Phenols such as Ph-2, Ph-3, and CA-20 were also shown to be very reactive in DMSO–Ac_2_O (yield > 97%). In contrast to alcohols, small phenols such as Ph-3 were mono- and di-methylthiomethylated by DMSO in the presence of Ac_2_O. According to Hayashi and Oda,^36^ phenols produced a higher yield of *ortho*-methylthiomethylated (MTM) products whereas phenols without an unsubstituted *ortho* position *e.g.* 2,6-dimethylphenol, gave *para*-alkylated products. Our results showed that varied yields of MTM products were observed in almost all phenols with an available *ortho* position in the benzene ring. The GC data presented in Fig. S1–S20,† suggest that isomeric mono-MTM and di-MTM products can be produced. For instance, Ox-9, a composition with *ortho*, *meta* and *para* positions, produced a mono-MTM product detected at retention times of 36.999, 37.362 and 37.639 minutes. Following results in the literature, we have proposed an *ortho*-MTM (33%), *para*-MTM (4.2%) and a *meta*-MTM (2.7%) for the compositions eluting at 36.999, 37.362, and 37.639 min, respectively. Thus, compositions with multiple available positions in the benzene ring (non-hindered) produced MTM products with higher yields. Exceptions were observed in vanillin and ethyl-vanillin (Ox-12 and Ox-11, respectively), where the aldehydes seem to hinder the MTM-attachment, leading to a preferable monoacetate product.

MTM by-products of the standards categorised as alcohols, catechols, and benzene diols were produced in negligible yields (<8%). Is interesting to note that an orange-coloured solution was observed for Ct-7 in DMSO–Ac_2_O whereas a dark brown solution was observed in the reaction of Ct-5, Ct-6, and Ct-8. Our results indicate that the former standards give similar reaction yields (89–92%) with products including monoacetate, diacetate and those of oxidation. Oxidation products were also observed in those samples producing orange and light-yellow solutions after reactions in DMSO–Ac_2_O (*e.g.* Ct-23, Ph-1, among others). 1,2-Phenylene diacetate was the major product of catechol in DMSO–Ac_2_O while a monoacetate (4-hydroxyphenyl acetate) and not the diacetate was the major acetate product observed in the reaction of hydroquinone. The difference in colour changes observed in catechols and benzene diols is believed to be related to the formation of the oxidised product (see for instance the mass spectrum obtained for Ct-5 DMSO–Ac_2_O at Rt 43.539 min in Fig. S5†). Hydroquinones and catechols play important role in chemical industries, biological processes, and environmental science. Our results indicate that a very distinctive and interesting reaction, likely corresponding to the oxidation of hydroquinone to the corresponding quinone, is possible in DMSO–Ac_2_O. Theoretical studies of the scale of oxidation potential of hydroquinones and catechols in DMSO indicates that the oxidation potential of hydroquinone is much smaller than that of catechol,^38^ however a more dedicated study of the reactions of catechols and hydroquinones in DMSO–Ac_2_O is necessary to clarify the reaction mechanisms involved. Such a study is out of the scope of this paper. In summary, alcohols and sterically hindered phenols presented the higher reaction yields in DMSO–Ac_2_O. While oxidation of alcohols was the main product observed for the standards categorised here as Ol, sterically hindered phenols were preferably methylthiomethylated in DMSO–Ac_2_O. Methylthiomethyl by-products were not observed in the reaction of catechols and vanillin. Finally, a negligible yield of the acetate products was observed in the reaction of both the standards classified as CA (containing carboxylic acids) and secondary alcohols.

#### Summary of reactions observed in lignin-derivatives

The hydroxyl moieties present in the standards analysed here can be classified as primary–secondary alcohols, phenols, sterically hindered phenols, and catechols (see summary in Table 2). Taken together, our results indicate that the hydroxyl moiety was selectively reactive with Ac_2_O, giving products in various yields in acetone or DMSO. It is noteworthy that the only side-reaction observed in this study corresponds to the esterification of the standards containing a carboxylic acid functional group, whereas other functional groups such as ester, ethers or ketones remained unchanged after the reaction. Although the reaction yields and products varied with the functional group profile of each standard, some general conclusions can be inferred from our results: (1) acetate products were observed in the reactions of non-hindered phenols, catechols and benzene diols in acetone–Ac_2_O, (2) primary and secondary alcohols only react efficiently in DMSO–Ac_2_O mixtures to produce an aldehyde or ketone, respectively, (3) catechols and benzene diols formed almost exclusively mono and di-acetate products in DMSO–Ac_2_O, and (4) a methylthiomethyl product was the major product observed in phenols with multiple *ortho*, *para*, and *meta* positions in the benzene ring. With those compositions that are less hindered (higher number of *ortho*, *para* and *meta* positions) producing di-MTM products with a higher yield.

**Table 2:** Summary of the major reactions of lignin derivative standards in acetone–Ac_2_O and DMSO–Ac_2_O mixtures. MTM: methylthiomethyl by-product. Carboxylic acids presented an additional esterification of the carboxylic acid site. Other functional groups (aldehydes, ketones, esters and ethers) were not reactive

Group		Acetone–Ac_2_O		DMSO–Ac_2_O	
		Main product	Yield (%)	Main products	Yield (%)
Ol (primary)	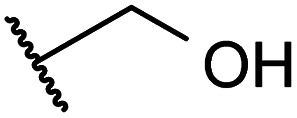	—	0.6	Aldehyde	∼97
Ol (secondary)	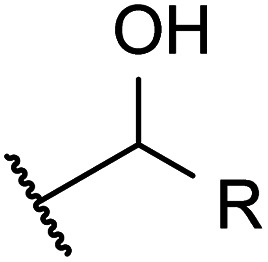	—	3.5^b^	Ketone	∼99.8
Ph	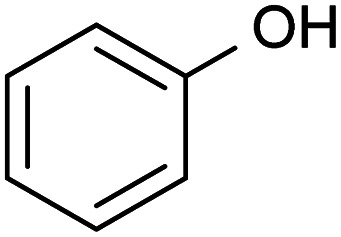	Monoacetate	62–35	Monoacetate MTM	69–49
Ph (hindered)	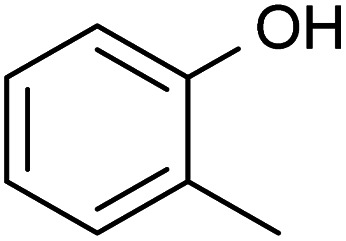	Monoacetate	∼18	MTM	∼97
Catechols and benzene diols	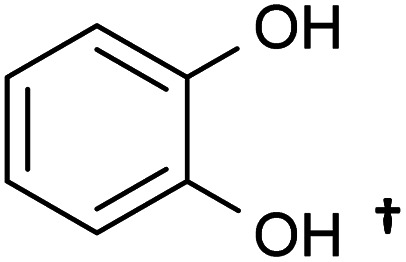	Monoacetate diacetate	∼65^a^	Monoacetate diacetate oxidation	92–89

a Excluding yields of Ct-25: catechol with a methyl group.

b A yield of 25% was observed for CA-20.

### Hydroxyl group of complex mixtures: bio-oil from lignocellulosic biomass

Direct infusion mass spectrometry measurements allow the simultaneous detection of all ionisable compounds in a single experiment. Mass spectrometers with a high resolving power can unambiguously discriminate individual elemental molecular compositions, however, additional chromatographic separation is needed to be able to discriminate between isomeric compounds.^39^ Therefore, the signal intensity of each detected *m*/*z*-value, may correspond to a combined signal contribution of ionised compounds with the same *m*/*z*-value but with a different functional group profile. Here, the derivatisation of the bio-oils in mixtures of acetone–Ac_2_O and DMSO–Ac_2_O is used to determine compositions containing a hydroxyl group. The results are summarised in Table 3 and the mass spectra are shown in Fig. S23–S24.†

**Table 3:** Summary of assignments and commonality of the bio-oil in different solvents obtained by direct infusion FTICR MS

Bio-oil		Acetone	Acetone–Ac_2_O	DMSO	DMSO–Ac_2_O
		A	B	C	D
Assignments	Total	4055	5471	4599	22 089
Mass range	(Da)	120–830	120–850	120–926	120–1200
Heteroatomic class total number (%)	O_o_[H]	4032 (>99)	5393 (>98)	4338 (94.3)	6260 (28.3)
	O	—	—	—	639 (2.9)
	O_o_S_s_[H]	—	—	—	15 060 (68.1)
	Other	23	78	261	130 (0.6)
Elemental contribution (%)^a^	Carbon	70	65	70	63
	Hydrogen	6.5	6	6.1	5.9
	Oxygen	24	29	24	24
	Sulfur	—	—	—	7.6
	Nitrogen	<0.2	<0.1	<0.1	<0.2
Lignin (%)	0.7 < H/C < 1.5	92	93	89	87
	0.1 < O/C < 0.67				
Commonality	Before/after reaction	**Acetone mixtures**		**DMSO mixtures**	
Common assignments		Common in A and B = 3569		Common in C and D = 3798	
Unique assignments		486	1902	801	18 291^b^

a Percentage contribution to total signal calculated with direct infusion FTICR data. The intensity of each MS was normalised to 100%.

b 14 800, 2852 and 639 corresponding to O_o_S_s_[H], O_o_[H] and O heteroatomic classes respectively.

#### Elemental composition profile of bio-oils before and after reaction in Ac_2_O

**Fig. 2:**
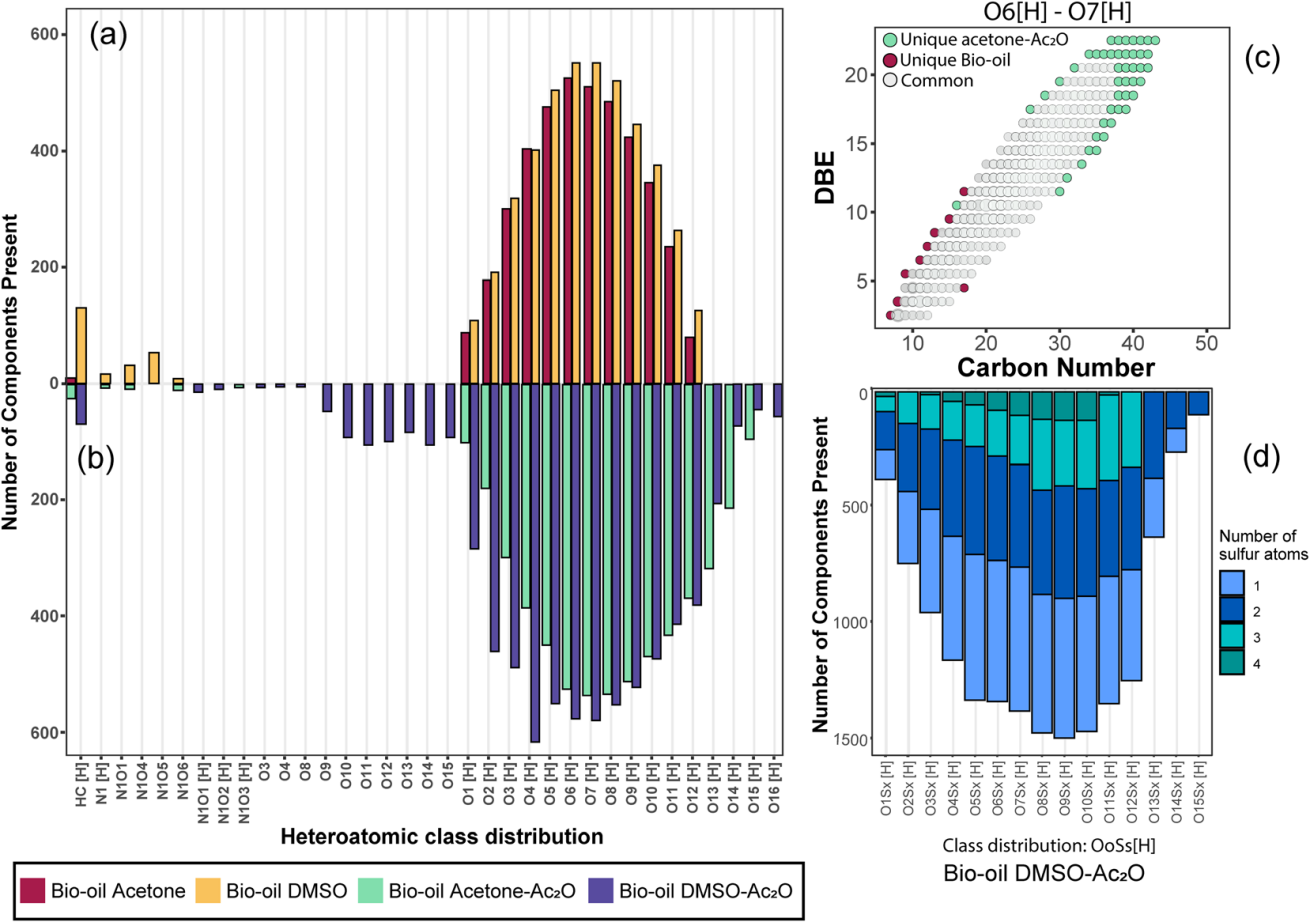
Heteroatomic class distribution of the bio-oil: (a) before reaction (b) after reactions in the presence of acetic anhydride. (c) DBE plot for classes O_6_[H] and O_7_[H] before and after derivatisation, common species represent overlapping molecular compositions, and (d) class distribution of the sulfur–oxygen containing species, detected only in the sample bio-oil DMSO–Ac_2_O.

A total of 4055 and 4599 elemental molecular assignments (including isotopologues) were detected for the non-derivatised bio-oils in acetone and DMSO, respectively. The assignments were sorted according to heteroatom class as shown in Fig. 2. As can be seen in Fig. 2(a), APCI operating in positive ion mode, enabled the detection of mainly protonated molecular compositions containing one to twelve oxygen atoms (classes O_1_[H] and O_12_[H], respectively), other heteroatoms such as nitrogen- and sulfur-containing species were not detected in the bio-oil before derivatisation. About 90% of these compositions have a H/C-ratio between 0.7–1.5 and an O/C-value between 0.1–0.67 (see van Krevelen diagram in Fig. S25,† and Table 3); this heteroatomic ratio is typically associated with lignin-derived chemical compounds.^29,40^

The elemental composition profile of the bio-oil after derivatisation in DMSO–Ac_2_O mixtures is very distinctive from that of the bio-oil blank, while some differences were also observed when the reaction was performed in acetone–Ac_2_O (see Fig. 2(b)). A shift towards higher oxygen-containing species was evident when the bio-oil was subjected to derivatisations in acetone–Ac_2_O, which is expected if acetate esters of phenols are formed by the addition of acetyl groups to the organic mixture. Unfortunately, the products from this reaction can overlap with a molecular composition already detected in the bio-oil blank (see Fig. 2(c)), henceforth a common molecular formula between the bio-oil before and after the derivatisation reaction cannot be discriminated with direct infusion experiments. A transformation of the hydroxyl group to a new chemical class is more advisable for direct infusion experiments, *e.g.* the addition of sulfur as described below. Despite this disadvantage, it was possible to detect 1835 unique molecular compositions in the bio-oil acetone–Ac_2_O. The former molecules correspond to acetate products of the reaction mainly assigned to O_8_[H]–O_15_[H] oxygen-containing heteroatomic classes. Considering the results observed in standard compositions, these molecular compositions are attributed to acetate products of non-sterically hindered phenols. The bio-oil elemental chemical compositions increased by five-fold after derivatisation in DMSO–Ac_2_O mixture. This is clear evidence of the diverse hydroxyl group profile present in the bio-oil. As can be seen in Fig. 2(b) the compositions detected in the bio-oil DMSO–Ac_2_O sample correspond to even- and odd-oxygen-containing molecular classes ([M + H]^+^ and M˙^+^, respectively), O_1–13_S_1–4_[H] (see Fig. 2(d)), and some other heteroatomic compositions in lower abundance. O_o_S_s_[H] heteroatomic classes (68.1% of the total signal contribution) correspond to methylthiomethyl by-products likely produced from the reaction of phenols with *ortho*, *para*, and *meta* positions (non-sterically hindered, see Fig. 2(d)) and guaiacol-like molecules (see products in Ph-1, Ph-2, and Ph-3). The synthesis of methylthiomethyl esters of carboxylic acids in DMSO have been previously reported by Yu *et al.*^41^ However, such a reaction was not observed in the CA-standards with our reaction conditions.

It is important to mention that the addition of sulfur after the reaction in DMSO–Ac_2_O introduces additional molecular compositions with a mass difference of 3.37 mDa corresponding to C_3_*vs.* SH_4_ (*e.g.*, C_20_H_16_O_4_[H] *vs.* C_17_H_20_O_4_S_1_[H], see Fig. S24†). A resolving power of 95 000 is needed to separate C_20_H_16_O_4_[H] from C_17_H_20_O_4_S_1_[H], thus ultrahigh resolution mass spectrometers such as FTICR MS are uniquely suited to characterise this sample. According to Table 3, the reactions in DMSO–Ac_2_O allowed the detection of 3491 oxygen-containing molecules that were not detected in the corresponding blank. Interesting, about 640 molecules were detected as odd-electron ions (M˙^+^) which were not detected in the blank. As shown in our previous section, DMSO–Ac_2_O changes the structure of the analyte to form products that were not observed in acetone–Ac_2_O. These molecules can be associated with products of oxidation of primary or secondary alcohols and the production of O-acetates.

#### Comparative evaluation of acetone–Ac_2_O and DMSO–Ac_2_O reactions

The derivatisations performed in lignin-derived compounds indicated that the reactions performed in a mixture of DMSO–Ac_2_O were more effective towards the transformation of the hydroxyl group. As shown in the following discussions, if a comparison of the reactions observed between acetone–Ac_2_O and DMSO–Ac_2_O mixtures is performed, it is possible to infer the presence of hindered phenols, non-hindered phenols, and alcohols.

**Fig. 3:**
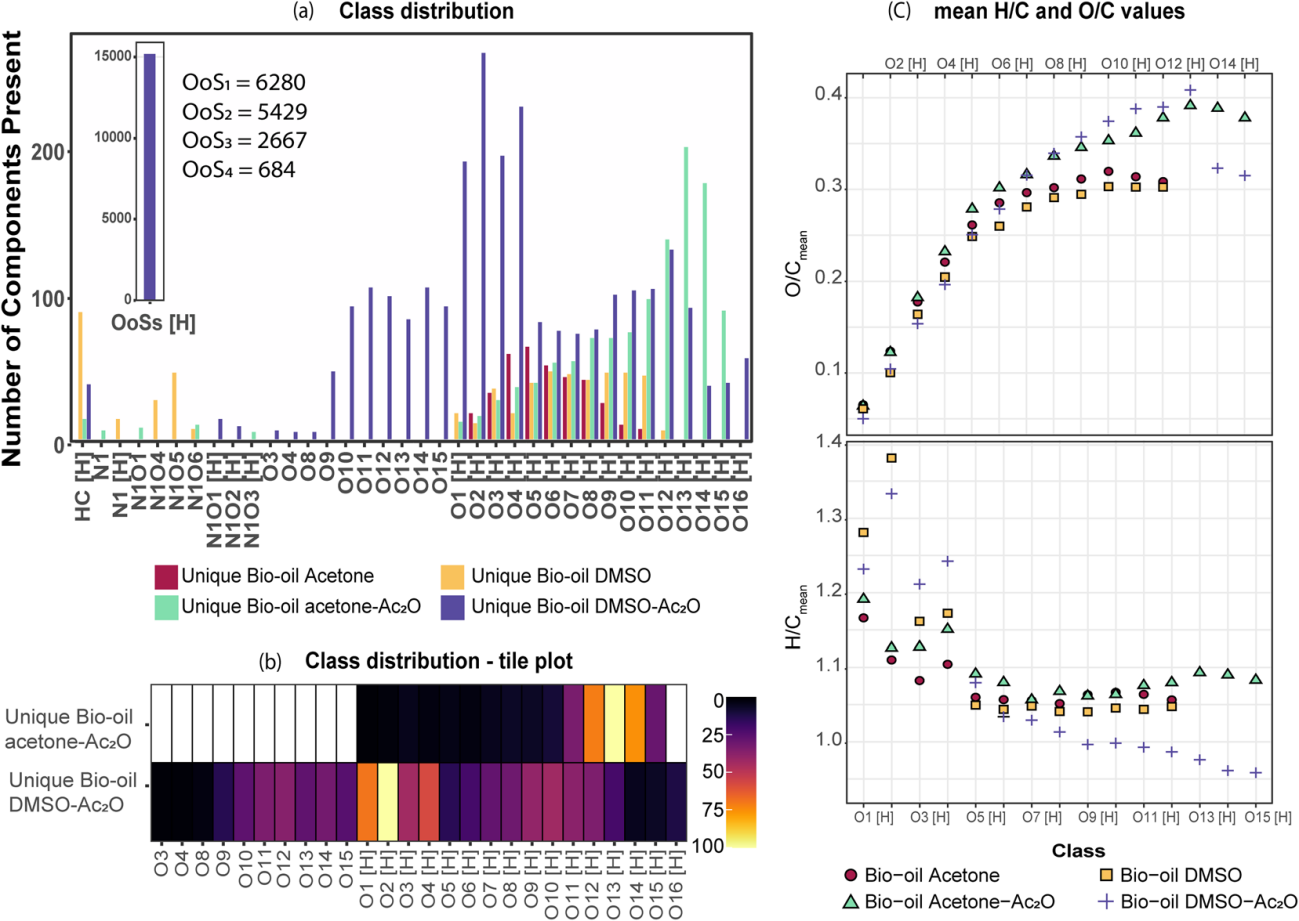
(a) Class distribution of unique assignments. (b) Tile plot of the unique oxygenated species detected in the reactions in acetone–Ac_2_O and DMSO–Ac_2_O. (c) Mean H/C and O/C values calculated based on the total number of compositions detected per heteroatomic class.

A comparison of the unique elemental molecular compositions of the reactions between acetone–Ac_2_O and DMSO–Ac_2_O is shown in Fig. 3 (see also Fig. S28†). In Fig. 3, common compositions between each reaction mixture and its corresponding blank have been excluded *e.g.* 3569 and 3798 common compositions found in the sample sets acetone *vs.* acetone–Ac_2_O, and DMSO *vs.* DMSO–Ac_2_O, respectively (see Table 3). As can be seen in Fig. 3a and b, a clear shift towards higher oxygen content species was observed in acetone–Ac_2_O mixtures. As previously discussed, this is a consequence of *o*-acetate formation of non-hindered phenols. In contrast, a higher dispersity of oxygenated compositions was detected in the reactions in DMSO–Ac_2_O. Odd-electron ions are uniquely detected in DMSO–Ac_2_O and correspond mainly to oxygenated species containing 10–15 oxygen atoms with a double bond equivalent between 14–22 (DBE plots can be found in Fig. S29†). The even-electron ions ([H]-class) detected in DMSO–Ac_2_O mixtures presented a double distribution, one with a distribution with O_1_[H] to O_4_[H] oxygenated compositions and another distribution corresponding to O_9_[H]–O_13_[H]. More than 15 000 ions corresponding to O_o_S_s_[H] heteroatomic class give us an indication of a high number of molecules containing phenols with, more likely, *ortho* and *para* positions.

The mean- H/C and O/C values per each heteroatomic class are shown in Fig. 3c. As can be seen in this figure, the O/C_mean_ of each class is similar between the blank samples (bio-oil acetone and bio-oil DMSO). In contrast, both bio-oils reacting in acetone–Ac_2_O and DMSO–Ac_2_O show an increased O/C_mean_ as the oxygen content in the molecule increases. This is a clear indication of the formation of acetate products after the reaction. The H/C_mean_ values of the sample bio-oil acetone–Ac_2_O remains very close to that of the blank bio-oil acetone for all the oxygenated classes. In contrast, most of the O_o_[H] classes in the DMSO samples presented a decreased H/C_mean_ value. A decrease in hydrogen-to-carbon value is an indication of oxidation of alcohols, a reaction that has been previously reported.^34^ This reaction was also observed in alcohols, some catechols, and benzene diol molecular standards as shown in Table 2 and Fig. 1. The presence of *p*-coumaryl alcohol, coniferyl alcohol and sinapyl alcohol, for instance, can lead to oxidation products of bio-oils reacting in DMSO–Ac_2_O mixtures.

**Fig. 4:**
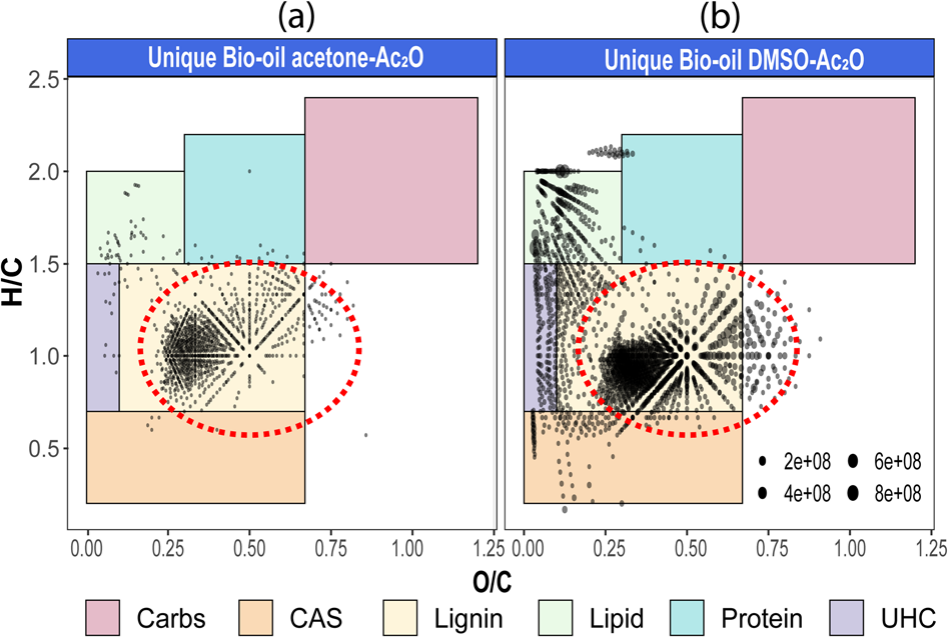
Van Krevelen plots of reactions performed in (a) bio-oil acetone–Ac_2_O, and (b) bio-oil DMSO–Ac_2_O. Only unique compositions of compositions when comparing acetone to acetone–Ac_2_O and DMSO to DMSO–Ac_2_O are plotted. Coloured boxes are used to indicate compositions classification. Here, Carbs: carbohydrates, CAS: condensed aromatic ring structures, UHC: unsaturated hydrocarbons. A red circle is used to indicate similar lignin-type compositions detected in both mixtures.

Van Krevelen diagrams of the unique species detected before and after derivatisation in each solvent are shown in Fig. 4 (see also van Krevelen diagrams per heteroatomic class in Fig. S29–S31†). As shown in Fig. 4, the compositions were classified by regions in which structures share common H/C and O/C elemental ratios.^40^ Comparison of the elemental compositions in both mixtures reveals that most of the unique compositions in acetone–Ac_2_O mixtures have elemental compositions similar to lignin, whereas lignin-type alongside with several unsaturated hydrocarbons (UHC), lipid-like, and condensed aromatic compositions were detected in the unique compositions after reactions in DMSO–Ac_2_O. As highlighted with a red circle in Fig. 3(b), odd-electron ion species and O_O ≥ 5_[H] are more likely *o*-acetate products of lignin-derivates (see also Fig. S31–32†), while several species assigned to O_1_[H]–O_4_[H] occupied the compositional space of UHC and lipid-like elemental compositions in DMSO–Ac_2_O mixtures. Huba *et al.*,^42^ have shown that aliphatic alcohols and aliphatic aldehydes, such as tetracosanol (C_24_H_50_O) and 1-octadecanal, respectively, are not efficiently ionised by APCI whereas aliphatic ketones presented a higher ionisation efficiency. Consequently, the lipid-like molecular compositions, with an H/C = 1.5–2, are more likely ketone products of the reactions in DMSO–Ac_2_O (Fig. 3). Lipophilic extractives such β-sitosterol (H/C = 2, O/C = 1/29) have been previously reported in wood-based fast pyrolysis.^43^ This indicates that the ketone products detected at a lipid-like compositional space in DMSO–Ac_2_O mixtures likely correspond to the oxidation of the secondary alcohol in sterol molecules. About 7% of the molecules detected uniquely in DMSO–Ac_2_O are in the compositional space associated with unsaturated hydrocarbons are probably oxidation products of moieties containing cyclic alcohols (cyclic ketones products).

The formation of an acetate product after the reaction will produce a molecule with a mass incremented by 42.010565 Da, which corresponds to the addition of C_2_H_2_O, this addition will in turn increase the O/C-value of the molecule. Similarly, the addition of multiple acetate products to a single molecule will also increase the O/C-value. For instance, the monoacetate and diacetate product of C_6_H_6_O_2_ (O/C = 2/6 = 0.333) will have an O/C-value of 0.375 (O/C = 3/8) and 0.4 (O/C = 4/10), respectively.

**Fig. 5:**
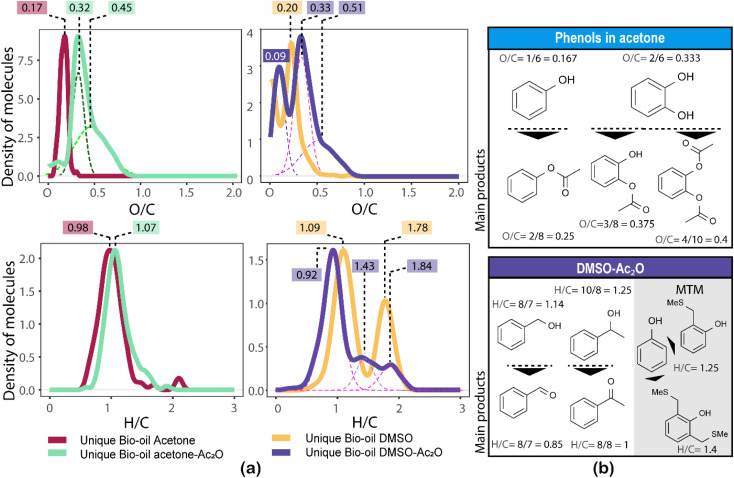
Left: Density of molecules along the H/C and O/C values corresponding to the unique oxygenated compositions. The mean values of the highest density areas are written at the top of the figures. Gaussian were fitted under the curves to calculate the mean values. Right: Suggested type of main products observed in each mixture.

Similarly, the oxidation of primary and secondary alcohols will reduce the H/C value of the molecules (see related examples in Fig. 5). Consequently, the density of molecules along the H/C and O/C axes will allow a quick visual comparison of the oxygen and hydrogen profile of the samples after derivatisation (see Fig. 5(a)). Two highly populated areas are observed at similar mean O/C-values for the bio-oil in both acetone–Ac_2_O and DMSO–Ac_2_O mixtures. The first distribution has a mean O/C-value of 0.32 and might correspond to mainly mono-acetate products while di-acetate products might be contributing to the density of molecules with a O/C-value of 0.45 and 0.51 in acetone–Ac_2_O and DMSO–Ac_2_O, respectively. The higher reaction yields observed in DMSO–Ac_2_O explains the relatively higher mean O/C-value observed for the reaction of bio-oil in this mixture. An additional high density of molecules located at a mean O/C-value of 0.09 was observed in DMSO–Ac_2_O, these compositions correspond to the contribution of species at the lowest O/C-values in the van Krevelen diagram shown in Fig. 4 (lipid-like and UHC-like molecular compositions).

The marginal increase in the H/C-value after the reactions in acetone–Ac_2_O can be a consequence of esterification side reactions analogous to the ones observed in CA-standards. In contrast, the density of unique molecules detected in DMSO–Ac_2_O are observed at a reduced H/C-value. Common monomers within lignin chemistry include vanillin alcohol (H/C = 1.25), homovanillyl alcohol (H/C = 1.33), veratryl alcohol (H/C = 1.33) and dihydroconiferyl alcohol (H/C = 1.4) are prone to oxidise in DMSO–Ac_2_O to form molecules with an H/C value between 1–1.2 (peak with a mean H/C = 0.92). The oxidation of lipid-like compositions and unsaturated hydrocarbons, which comprise a mixture of aliphatic and aromatic moieties, are more likely contributing to the density of molecules at a mean H/C = 1.84 and 1.43 respectively. Methylthiomethyl products are mostly located within the lignin-type compositional space in van Krevelen diagrams (see Fig. S32–S35†). This indicates the presence of molecules containing phenols such as the ones shown in Fig. 5(b).

### Semi-quantitative speciation of the hydroxyl group

FTICR MS capabilities allows the simultaneous detection and unique assignment of thousands of individual elemental compounds in a single mass spectrum. Several factors limit the quantification by MS: ionisation yields differ between the different compounds, matrix effects, unknown chemical structures, selective solubility, alongside an undefined number of isomeric contributions per individual elemental compound.^44,45^ Thus, considering these factors only semi-quantification can be achieved. It is important to mention, however, that oxygenated compositions as the ones presented in Table 1 have been shown to be ionizable by positive ion-mode APCI.

The elemental compositions of each sample are compared to deliver a semi-quantitative speciation of the hydroxyl group profile within the sample. A Venn diagram contained in Table 4, is used to illustrate such comparison. The area of the circles has been re-sized to correspond to the total number of oxygenated compositions detected in each sample *i.e.*, 4032 (acetone blank), 5383 (acetone–Ac_2_O), 4338 (DMSO blank) and 6899 (DMSO–Ac_2_O).

**Table 4:** Semi-quantitative analysis of the hydroxyl group content in the bio-oil. The oxygenated species defined as total O_o(solvent–AC_2_O)_ in the table was used to calculate the percentage in each solvent^b^

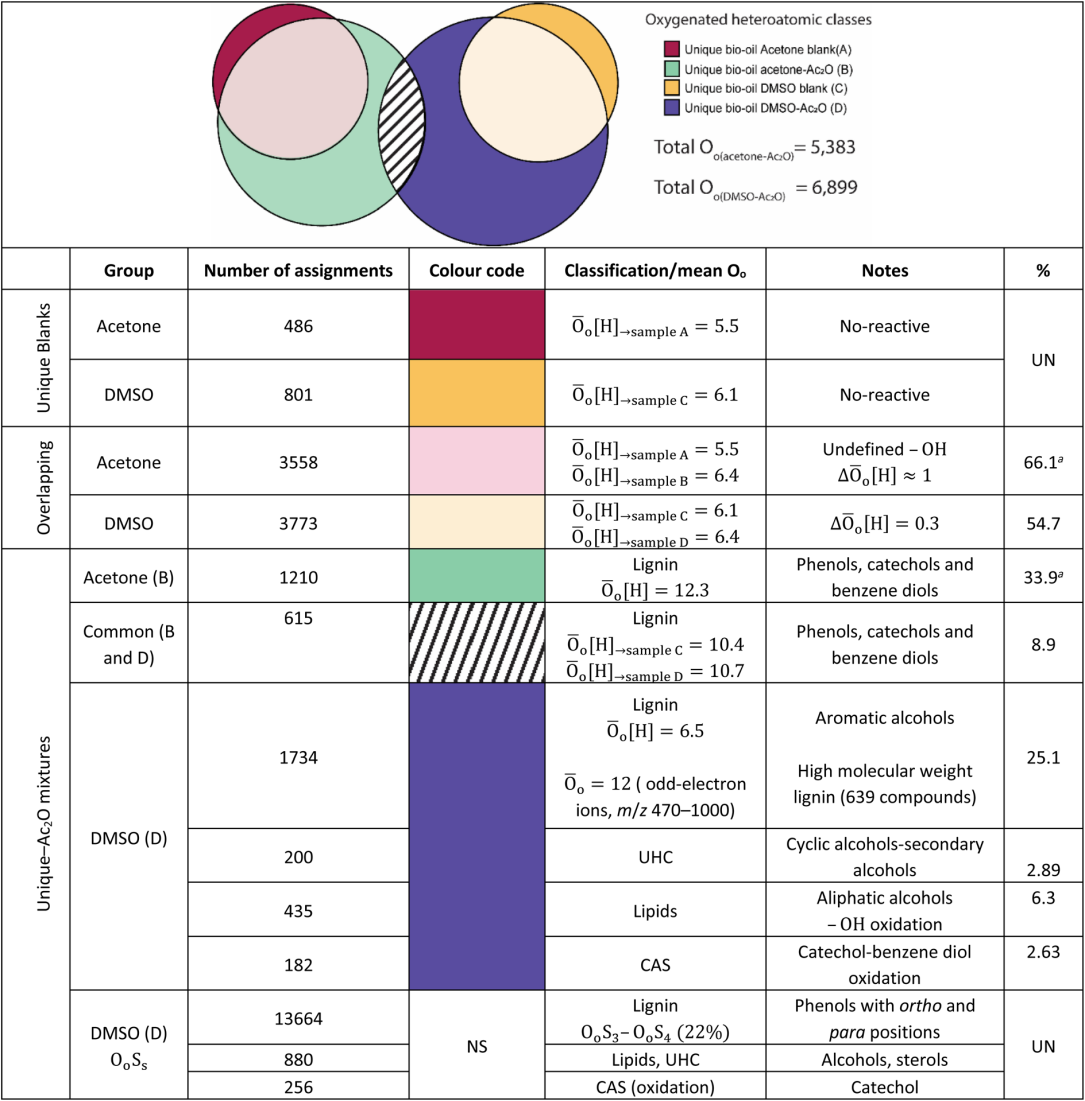

a Percentage for sample bio-oil acetone–Ac_2_O.

b Ō_o_ = mean O_o_ class_Normalised intensity weighted_, NS: not-shown in Venn diagram, UN: undefined.

As can be seen in Table 4, a significant number of compositions overlapped with a composition already detected within the blank. The percentage of non-reactive and overlapping material is then undefined with the current experimental data. It is interesting to note that the mean oxygen content is increased by about one oxygen atom when the derivatisation is performed in acetone–Ac_2_O, which indicates that mono-acetate products are contributing to the total intensity within the overlapping compositions. In comparison, the overlapping compositions in DMSO did not present a significant increase of the oxygen content after derivatisation.

The separation of the overlapping products and reactants will require the use of a separation technique before ion detection that are currently out of the scope of this study. Our data indicates that the hydroxyl group is mostly present in phenolic-like containing molecules (∼34%), followed by aromatic alcohols (benzyl alcohol-type, ∼25%). Fewer unique compositions corresponding to aliphatic alcohols (6.3%) and primary secondary alcohols, including cyclic alcohols (2.89) were also detected in DMSO–Ac_2_O mixtures. The percentage of phenol-like compositions falls close to the range typically reported for phenol oligomers in pyrolysis bio-oils, 26–33%,^46,47^ similarly 2–5% of alcohols have been previously reported in literature. Our method then also provides unique insights on the distribution of aromatic alcohols, aliphatic and cyclic alcohols in complex mixtures, information that is currently scarce in literature.

Our data also present clear evidence of the diversity of *ortho*, *para*, and *meta* positions in phenolic compositions. A semi-quantitation of these compositions is, however, more difficult. Firstly, an attachment of up to two methylthiomethyl chains to the *ortho*, *para*, or *meta* positions was observed within the reaction products of the standards, hence, the quantitation of MTM chain attachment is difficult. Secondly, the fragmentation of the MTM chain to produce a O_o_S_1_ elemental composition was observed for the standard compositions (see for instance Fig. S3†), although the corona current was reduced for the acquisition of the UHRMS data of the bio-oils (see Method section), the degree of fragmentation of O_o_S_s_ compositions is difficult to estimate. Thirdly, competition between formation of MTM and ketones can be observed (*e.g.*, Ol-16 and Ol-27). Finally, bio-oil compositions were characterised by a high-oxygen content in a single elemental molecule, therefore a combination of reactions cannot be discriminated. Thus, the presence of O_o_S_s_ compositions is used as a qualitative metric of the distribution of *ortho*, *para* and *meta* positions in phenols. According to Fig. 3, S_1_ to S_4_ oxygenated molecular compositions (O_o_S_1–4_[H]) were detected. Compositions with at least two *ortho*, *para* or *meta* positions will yield a O_o_S_1_ and O_o_S_2_ elemental composition (78%, calculated data shown in Fig. 3), this indicates the high number of moieties containing phenols similar to Ph-1, Ph-2 and Ph-3 (hindered phenols). Notice that catechols, benzene diols, and phenols containing an aldehyde (such as vanillin) are more likely transformed to acetate products and, therefore, a lower signal contribution from these compositions to the O_o_S_s_ heteroatomic classes is expected. Alkylated phenols have been previously reported by Garcia-Perez *et al.*, in 2007.^48^ Examples include but are not limited to 2-methylphenol, 3,4-dimethylphenol, 3-methyl-1,2-benzenediol, and 3,4-diehtylphenol. These phenols can be transformed to MTM products in DMSO–Ac_2_O. About 22% of sulfur-oxygenated containing classes contain three to four sulfur atoms. The attachment of more than two MTM chains was not observed within the products observed in the reaction of the standards, is then likely that the bio-oil is composed of an arrangement of monomers such as *p*-coumaryl alcohol and coniferyl alcohol. Oligomers containing *p*-coumaryl end groups (two *ortho* positions) are likely precursors of the O_o_S_3–4_[H] molecular compositions whereas coniferyl alcohol end groups are more likely precursors of compositions containing one to two sulfur–oxygen containing species (78%). This result is in agreement with previous literature.^49^

According to the data discussed in previous sections, acetone–acetic anhydride reactions and dimethyl sulfoxide–acetic reactions can be used to provide unique and distinctive insights into the hydroxyl functional group profile in complex mixtures. In summary, alcohols are non-reactive in acetone–Ac_2_O mixtures, phenols with *ortho* or *para* positions are mostly transformed to an MTM-product in DMSO–Ac_2_O, non-hindered phenols reacted in both mixtures, and alcohols oxidised when reacting in DMSO–Ac_2_O mixtures. The quantification of the hydroxyl group in individual moieties of a complex mixture will require the separation of each reactive chemical within the bio-oil, calibration curves, authentic standards covering the mass range of detection (4000–5000, elemental compositions were detected), and measuring ionisation response against concentration. Individual isomers are, however, not discriminated by direct infusion mass spectrometry and thus hyphenated techniques such as gas chromatography, GC × GC, and liquid chromatography, or alternatively, ion mobility^50–52^ can be used to deliver a more detailed insight of the hydroxyl group profile. Future work will focus on the use of chemical derivatisations combined with hyphenated mass spectrometry to allow the separation of isomeric compositions and overlapping reactant/products elemental molecular compositions.

## Conclusions

In the first part of this research, derivatisations using acetic anhydride in different solvents were employed to evaluate the reactivity of the hydroxyl groups in lignin-representative standards. The observed reactions are summarised as follows:

•Reactions in acetone–Ac_2_O: (1) mono and di-acetate products of phenols without saturated side chains with acceptable yields (65–35%) were observed, (2) primary and secondary alcohols were essentially non-reactive (yield < 3.5%), and (3) low reaction yields (<18%) were observed in sterically hindered phenols.

•Reactions in DMSO–Ac_2_O: (1) primary and secondary alcohols formed aldehyde or ketone products, respectively, with a high yield (97–99.8%) (oxidation reactions), (2) hindered phenol compounds such as o-cresol and guaiacol formed primarily a methylthiomethyl product (–CH_2_SCH_3_) with about 92% yield, and (3) phenol, catechol and benzene diol molecules formed primarily mono and di-acetate products.

•The standards were essentially non-reactive in MeOH–Ac_2_O mixtures.

Thus, the reactions in both acetone and DMSO, presented a high chemo-selective transformation of the hydroxyl group and DMSO–Ac_2_O reactions are advisable when higher yield reactions of the hydroxyl group are required.

The derivatisations combined with direct infusion ultrahigh resolution mass spectrometry were used to pinpoint elemental compositions containing a hydroxyl group in a bio-oil. Our results show that about 2000 and 18 400 new elemental compositions were detected in the bio-oil after derivatisations in acetone–Ac_2_O and DMSO–Ac_2_O mixtures, respectively. Those compositions correspond to reaction products from chemical moieties containing at least one hydroxyl group. The bio-oil acetone–Ac_2_O presented unique compositions in the compositional space corresponding to lignin-like structures, which indicates the formation of acetate products of non-hindered phenols. The extraordinary increased number of compositions of the bio-oil DMSO–Ac_2_O sample is clear evidence of the diverse hydroxyl group profile present in the bio-oil. DMSO–Ac_2_O reactions have the unique advantage of transforming the hydroxyl group to a new chemical class, *e.g.*, oxygen-containing species, O_o_, to heteroatomic classes corresponding to O_o_S_s_, species that can be easily separated by FTICR MS. Our results indicate that about 15 000 elemental compositions correspond to the methylthiomethyl product (O_o_S_s_-heteroatomic classes) likely corresponding to phenolic moieties with *ortho* and *para* positions. Additionally, unique compositions occupying the compositional space of lipid-like and UHC-like structures might correspond to oxidised reactions that uniquely occur in DMSO–Ac_2_O mixtures, confirming the presence of primary and secondary alcohols, including cyclic alcohols, within the chemical moieties found in a bio-oil. A semi-quantitative analysis indicates that about 34% and 25% of the new elemental compositions detected after reaction with Ac_2_O correspond to the transformation of phenolic-like and aromatic-alcohols, respectively.

The combination of the chemoselective transformation of a functional group in conjunction with ultrahigh resolution mass spectrometry can be used as a qualitative metric of the functional group profile of complex mixtures such as bio-oils. The production of fine chemicals and hydrocarbons from renewable sources such as bio-oils, relies on more efficient deoxygenation routes. Knowledge about the hydroxyl group profile of bio-oils, in particular the possible content of hindered and non-hindered phenols can be beneficial to predict the contribution of specific products if bio-oils are co-processed or upgraded *via* acidic catalysts.

## Data availability

The research data (and/or materials) supporting this publication can be accessed at http://wrap.warwick.ac.uk/

## Conflicts of interest

There are no conflicts to declare.

## Supplementary Material

RA-013-D3RA02779A-s001

RA-013-D3RA02779A-s002
