# Supplementary material for: Chemoselective derivatisation and ultrahigh resolution mass spectrometry for the determination of hydroxyl functional groups within complex bio-oils
Source: RSC Adv. 2023 Jun 12;13(26):17727–41. doi: 10.1039/d3ra02779a (PMC10259504; doi:10.1039/d3ra02779a)
Supplement: RA-013-D3RA02779A-s002 [file RA-013-D3RA02779A-s002.pdf]

Electronic Supplementary Material (ESI) for RSC Advances.
This journal is © The Royal Society of Chemistry 2023

Supporting information

Chemoselective derivatisation and ultrahigh resolution mass spectrometry for the determination of hydroxyl functional groups within complex bio-oils

Diana Catalina Palacio Lozano,[1*] Hugh E. Jones,[1] Mark P. Barrow,[1] and Martin Wills[1]

[1] Department of Chemistry, University of Warwick, Coventry, CV4 7AL, UK.

E-mail: d.palacio-lozano@warwick.ac.uk

Contents

1.1 Description of standards 1
1.2 Chromatograms 3
.... 3
1.3 Reaction mechanisms 23
1.4 Reactions 24
1.5 Mass spectra 26
1.6 Van Krevelen and DBE plots 28

1.1 Description of standards

Table S 1. Detailed list of standards

Name	CAS-No	Id	Vendor	Purity (%)	Structure
2-methoxyphenol	90-05-1	Ph-1	Thermo Fisher	99	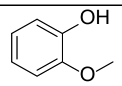
2,4-dimethylphenol	105-67-9	Ph-2	Acros Organics	98	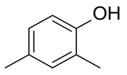
o-cresol	97-48-7	Ph-3	Acros Organics	99	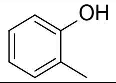
4-ethylphenol	123-07-9	Ph-4	Sigma-Aldrich	97	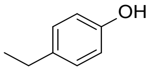
hydroquinone	123-31-9	Ct-5	Sigma-Aldrich	99	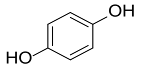
1-(2,5-dihydroxyphenyl)propan-1-one	938-46-5	Ct-6	Abcr	97	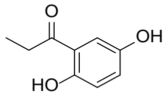

5-methylbenzene-1,3-diol	504-15-4	Ct-7	Sigma-Aldrich	98	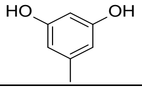
pyrocatechol	120-80-9	Ct-8	Alfa Aesar	97	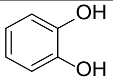
ethyl 2-hydroxybenzoate	118-61-6	Ox-9	Abcr	97	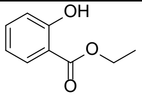
1-(3-hydroxy-4-methoxyphenyl)ethan-1-one	6100-74-9	Ox-10	Sigma-Aldrich	97	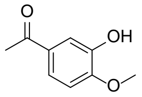
3-ethoxy-4-hydroxybenzaldehyde	121-32-4	Ox-11	Supelco	98	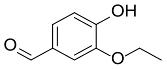
4-hydroxy-3-methoxybenzaldehyde	121-33-5	Ox-12	Sigma-Aldrich	98	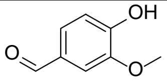
3-(hydroxymethyl)phenol	620-24-6	Ol-13	Abcr	97	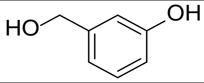
chroman-4-ol	1481-93-2	Ol-14	Alfa Aesar	97	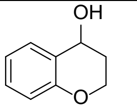
1-phenylethan-1-ol	98-85-1	Ol-15	Sigma-Aldrich	98	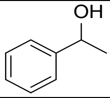
2-phenylethan-1-ol	60-12-8	Ol-16	Sigma-Aldrich	98	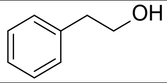
3-cyclohexylpropan-1-ol	1124-63-6	Ol-17	Thermo Fisher	99	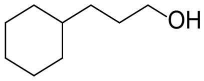
2-hydroxy-3-phenylpropanoic acid	828-01-3	Ca-18	Sigma-Aldrich	97	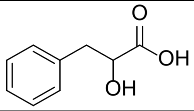
2-(4-hydroxyphenyl)propanoic acid	938-96-5	Ol-19	Abcr	98	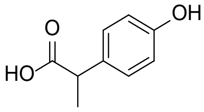
4-hydroxy-3,5-dimethylbenzoic acid	4919-37-3	Cas-20	Abcr	98	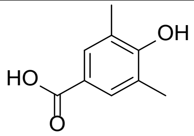
Bio-oil	1207435-39-9	BO			

1.2 Chromatograms

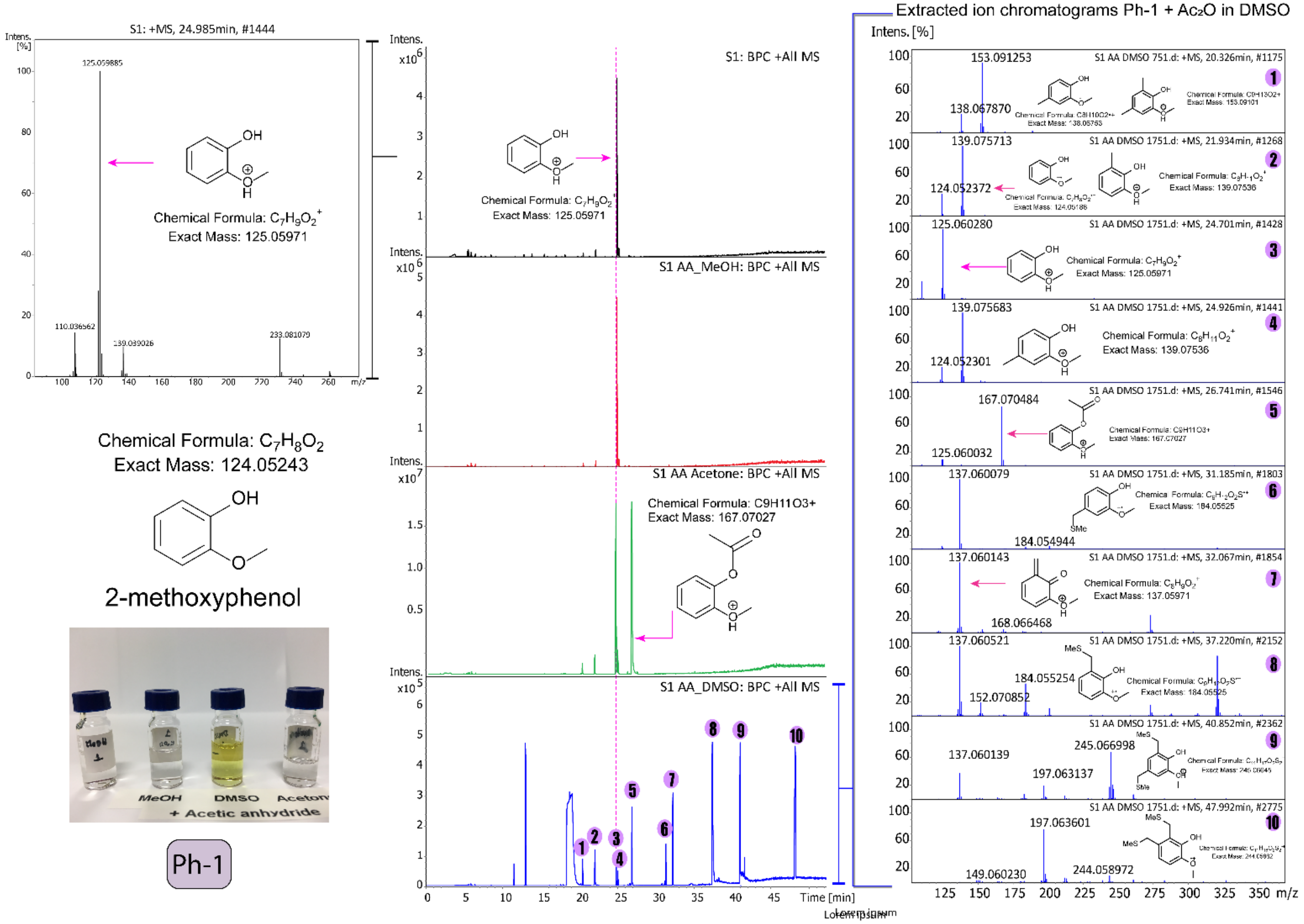

Fig. S1 Centre: chromatograms of Ph-1 without reaction (black), left: extracted MS of raw standard, right: MS at different RT for the reaction in DMSO-AC₂O. A photo of the samples is embedded on the left-bottom of the figure. Suggested structures have been embedded in the figure.

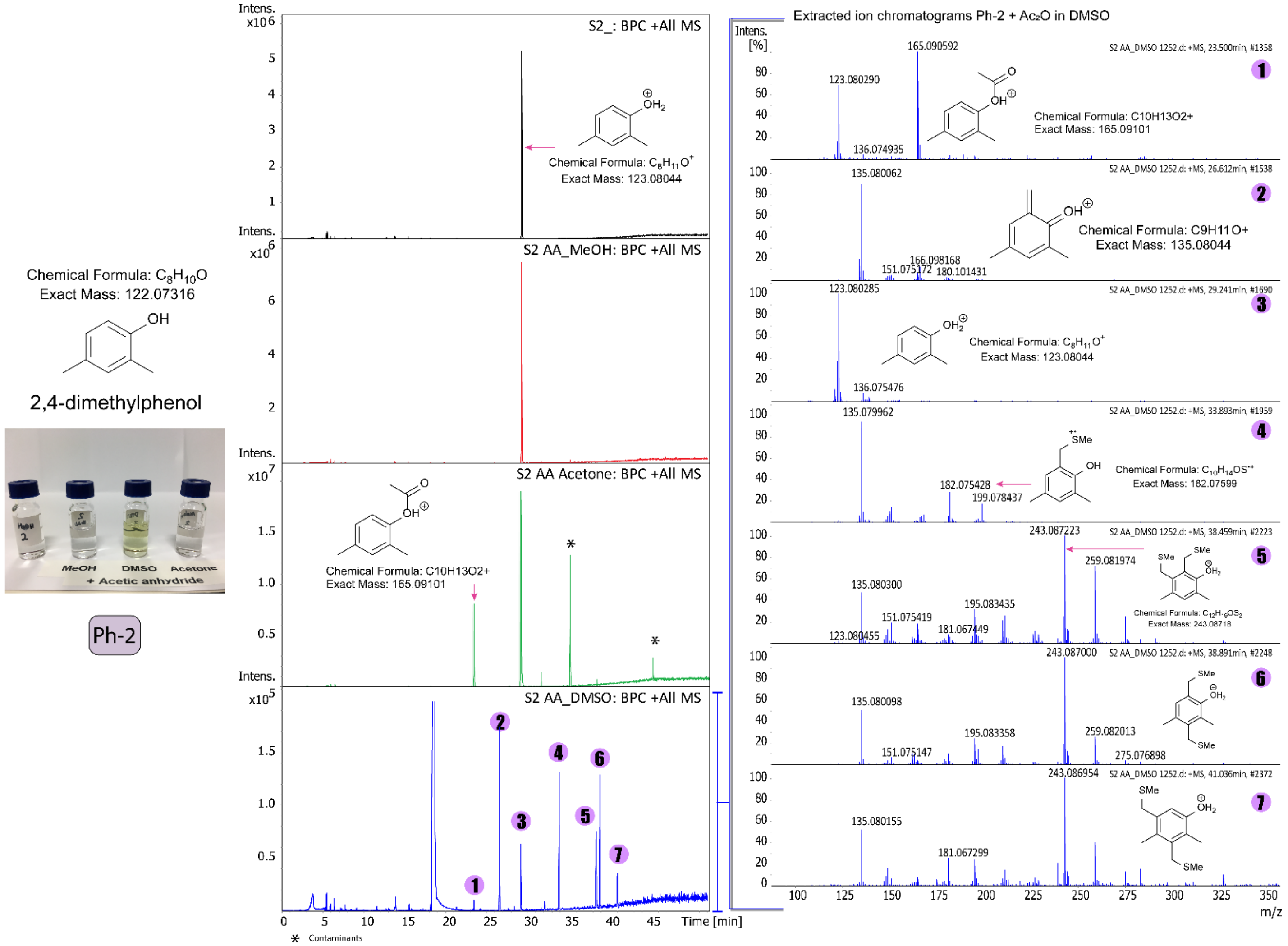

Fig. S2 Centre: chromatograms of Ph-2 without reaction (black), right: MS at different RT for the reaction in DMSO-AC₂O. A photo of the samples is embedded on the left-bottom of the figure. Suggested structures have been embedded in the figure.

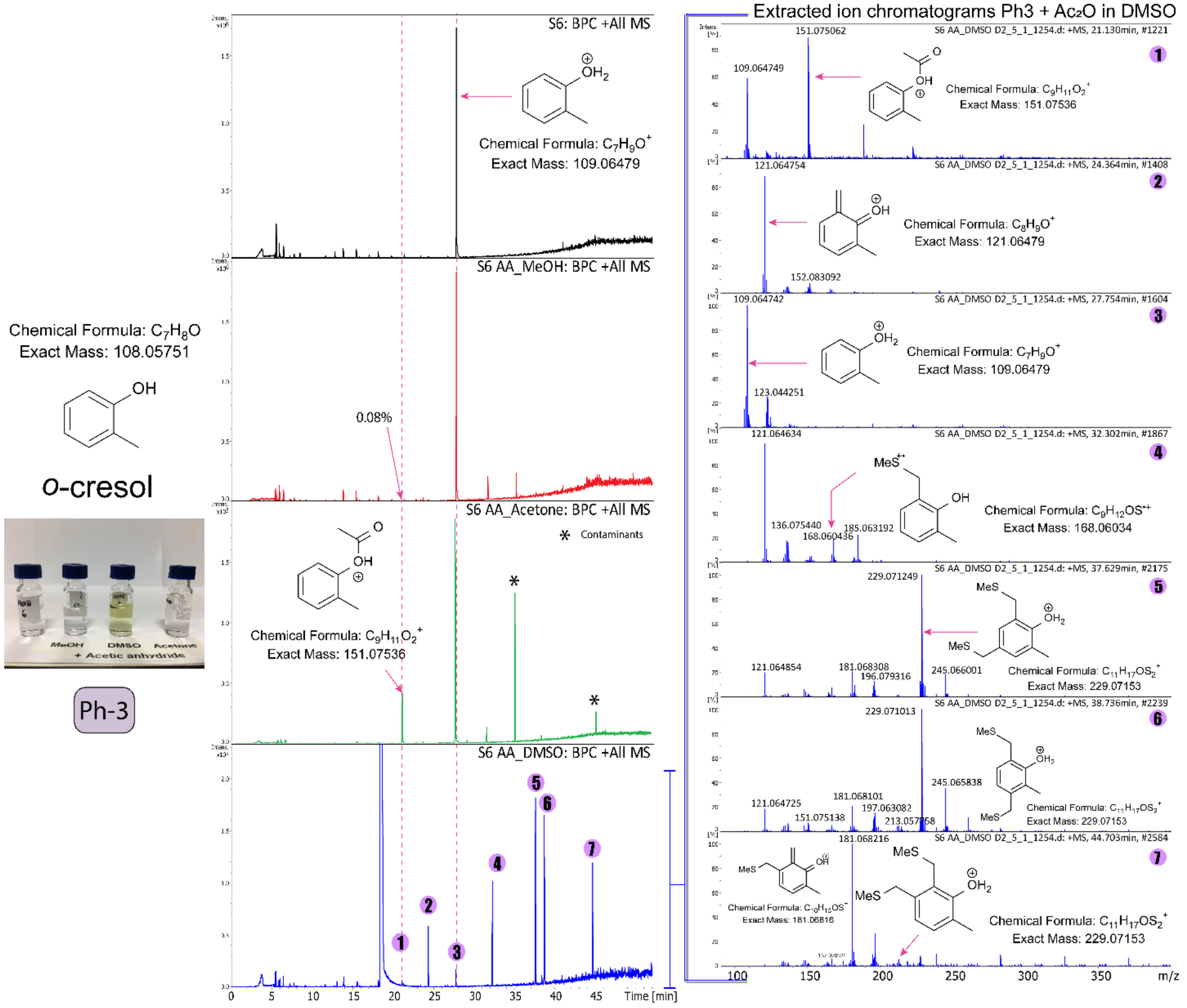

Fig. S3 Centre: chromatograms of Ph-3 without reaction (black), right: MS at different RT for the reaction in DMSO-AC₂O. A photo of the samples is embedded on the left-bottom of the figure. Suggested structures have been embedded in the figure.

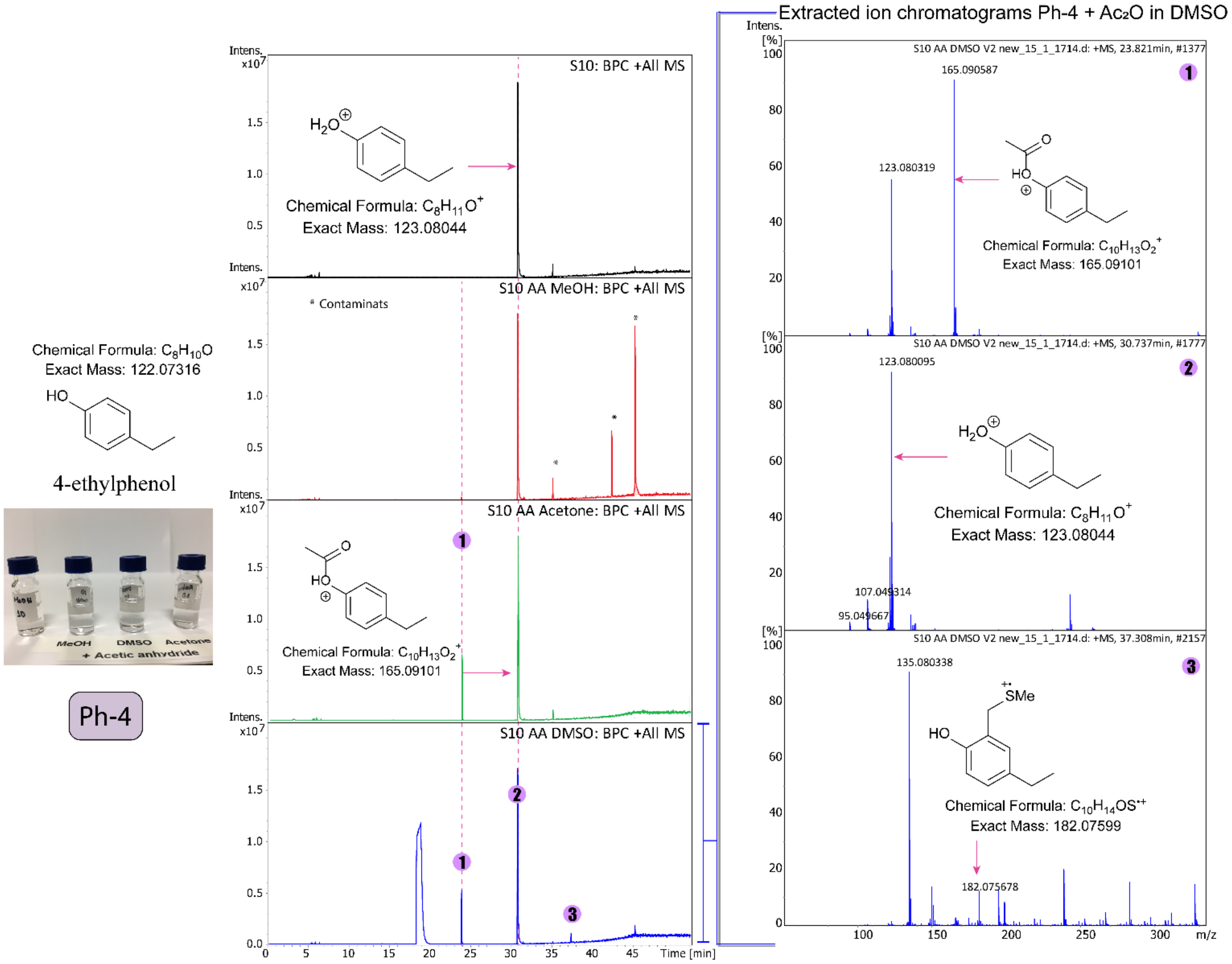

Fig. S4 Centre: chromatograms of Ph-4 without reaction (black), right: MS at different RT for the reaction in DMSO-AC$_2$O. A photo of the samples is embedded on the left-bottom of the figure. Suggested structures have been embedded in the figure.

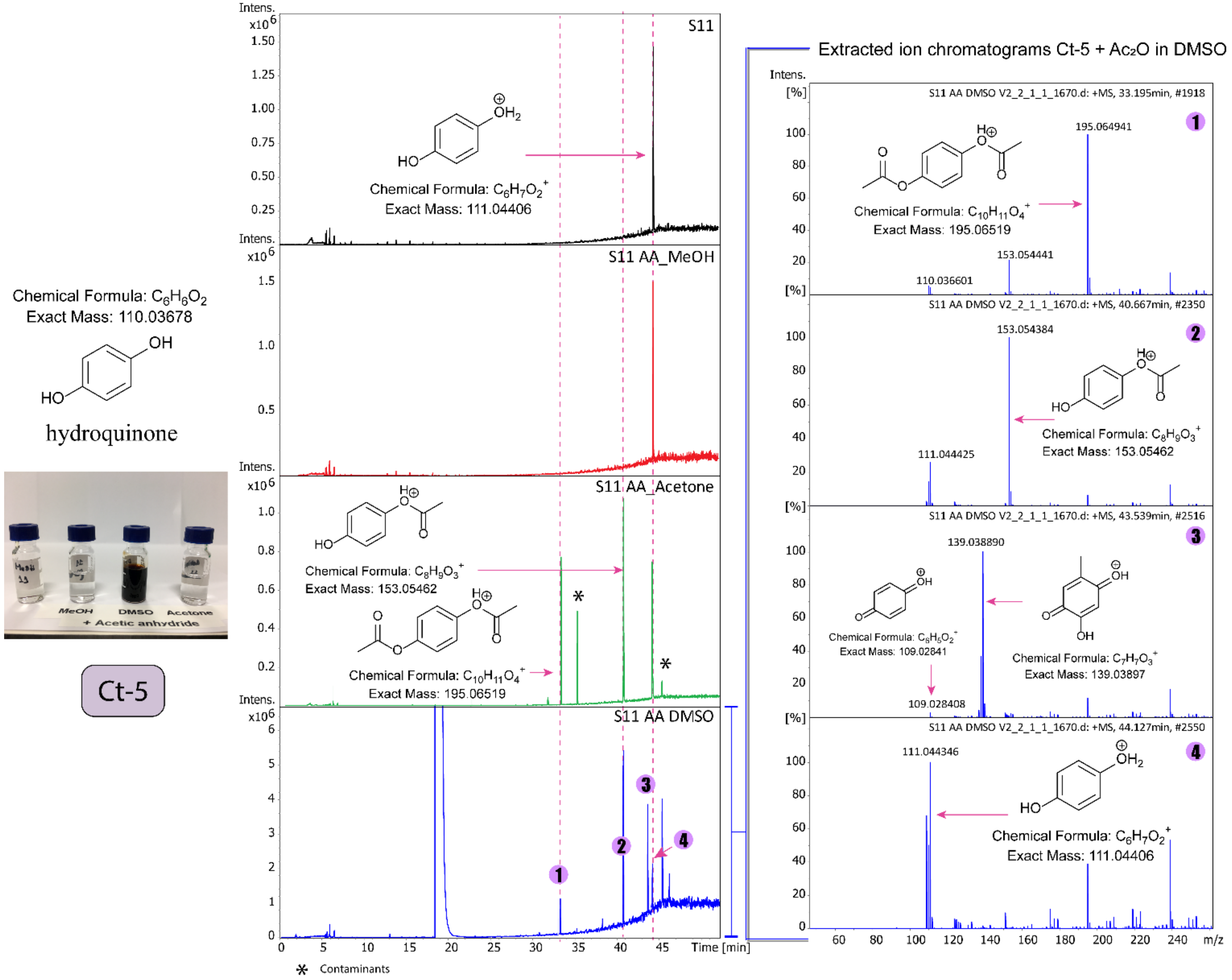

Fig. S5 Centre: chromatograms of Ct-5 without reaction (black), right: MS at different RT for the reaction in DMSO-AC$_2$O. A photo of the samples is embedded on the left-bottom of the figure. Suggested structures have been embedded in the figure.

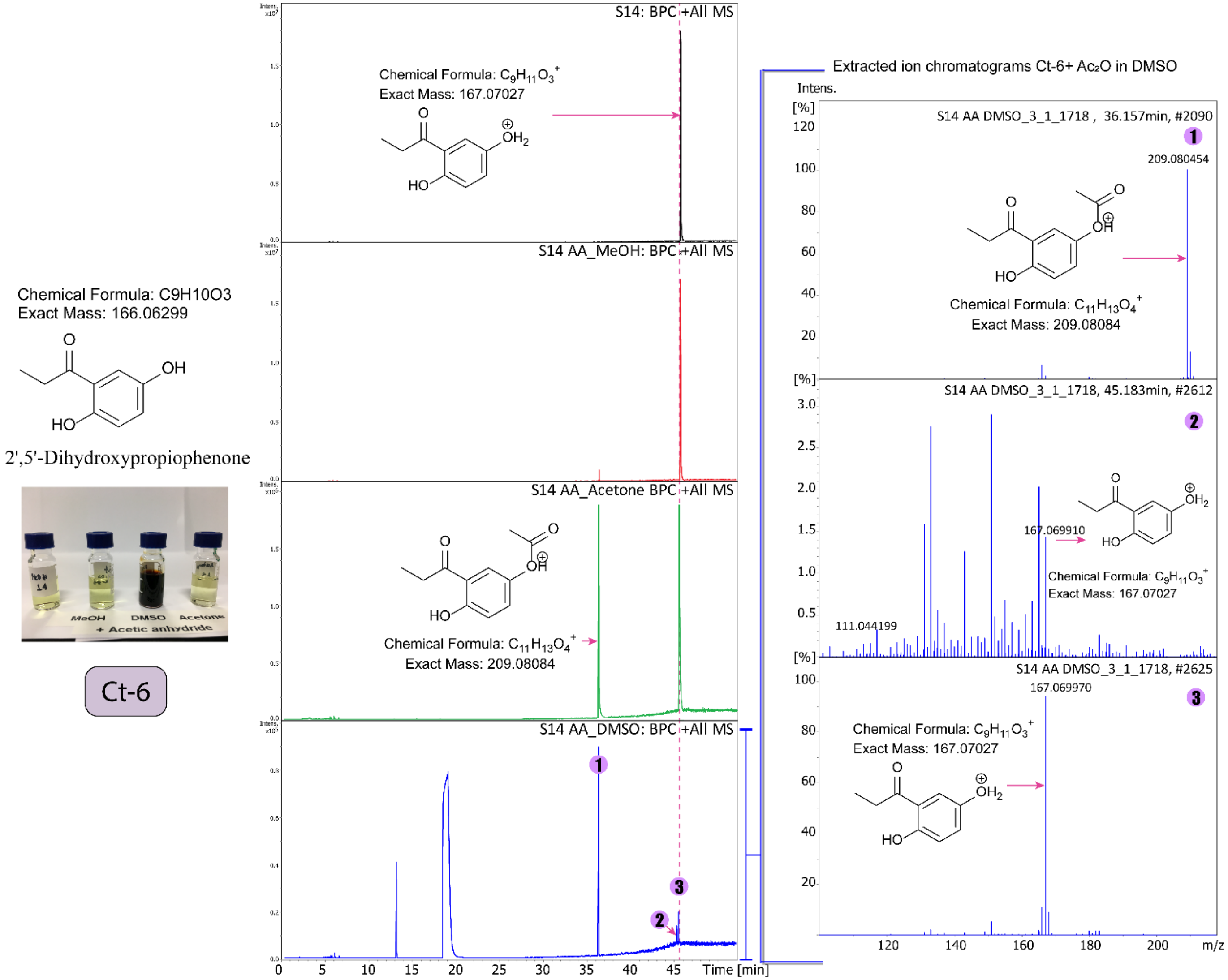

Fig. S6 Centre: chromatograms of Ct-6 without reaction (black), right: MS at different RT for the reaction in DMSO-AC₂O. A photo of the samples is embedded on the left-bottom of the figure. Suggested structures have been embedded in the figure.

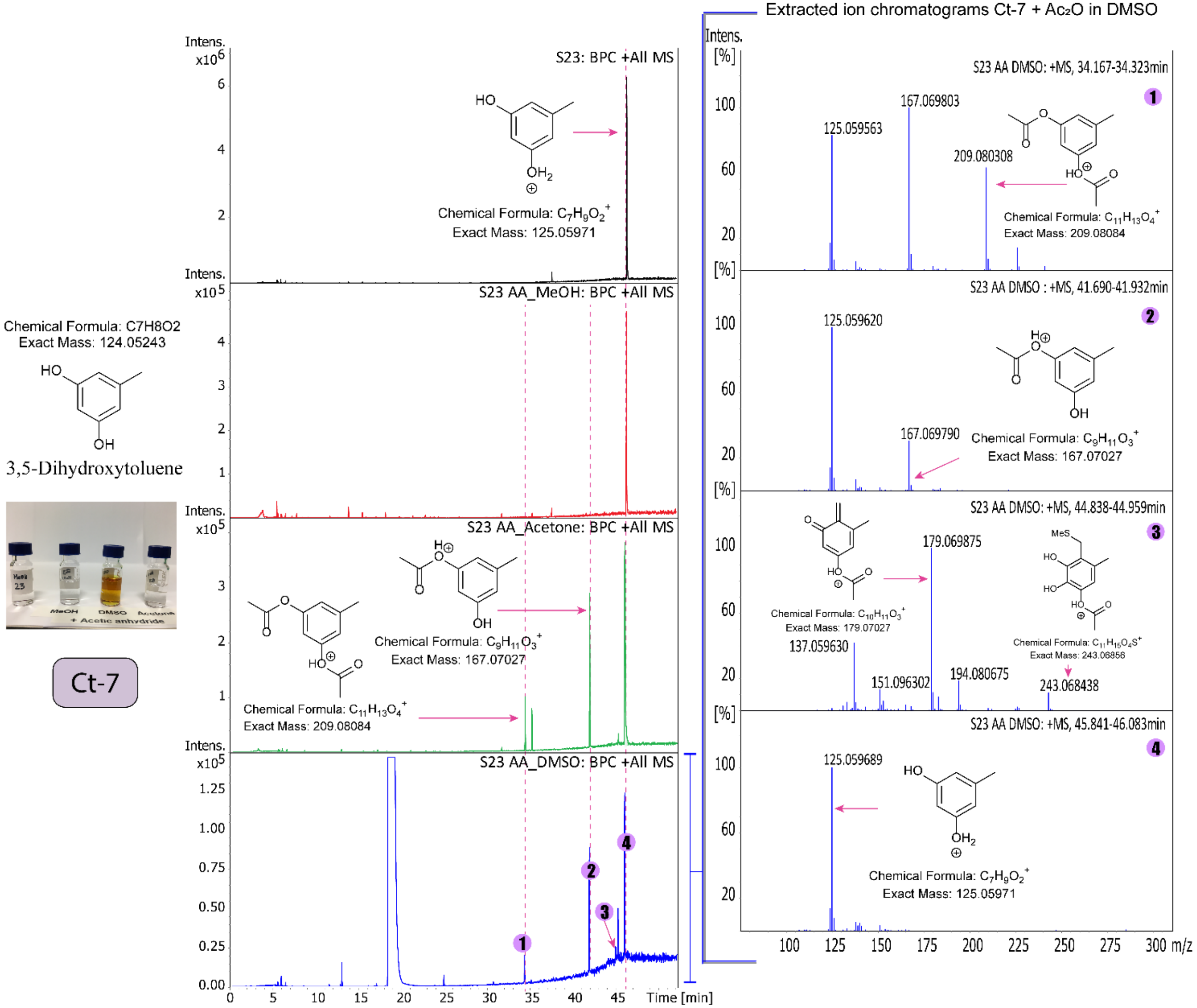

Fig. S7 Centre: chromatograms of Ct-7 without reaction (black), right: MS at different RT for the reaction in DMSO-AC₂O. A photo of the samples is embedded on the left-bottom of the figure. Suggested structures have been embedded in the figure.

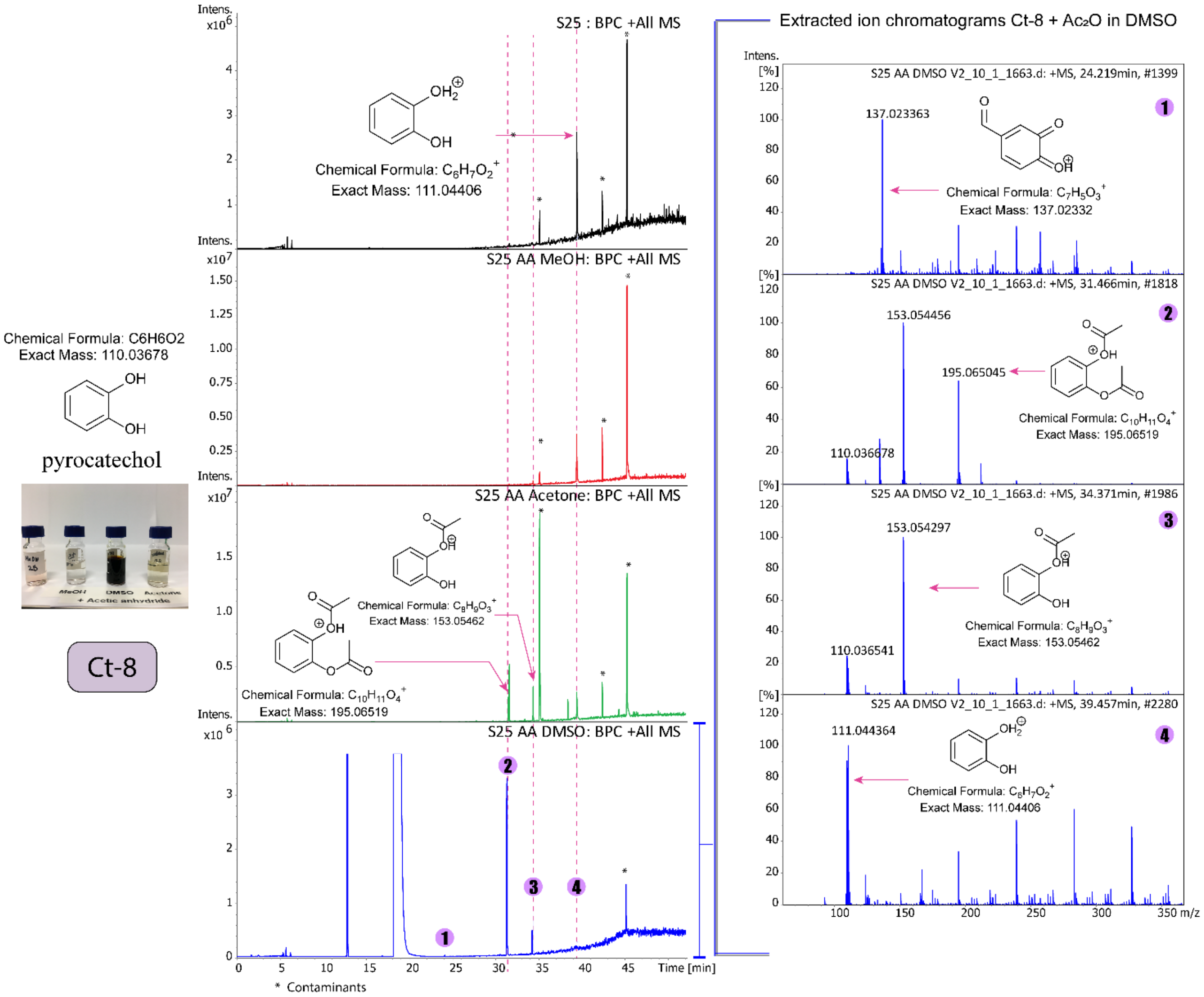

Fig. S8 Centre: chromatograms of Ct-8 without reaction (black), right: MS at different RT for the reaction in DMSO-AC$_2$O. A photo of the samples is embedded on the left-bottom of the figure. Suggested structures have been embedded in the figure.

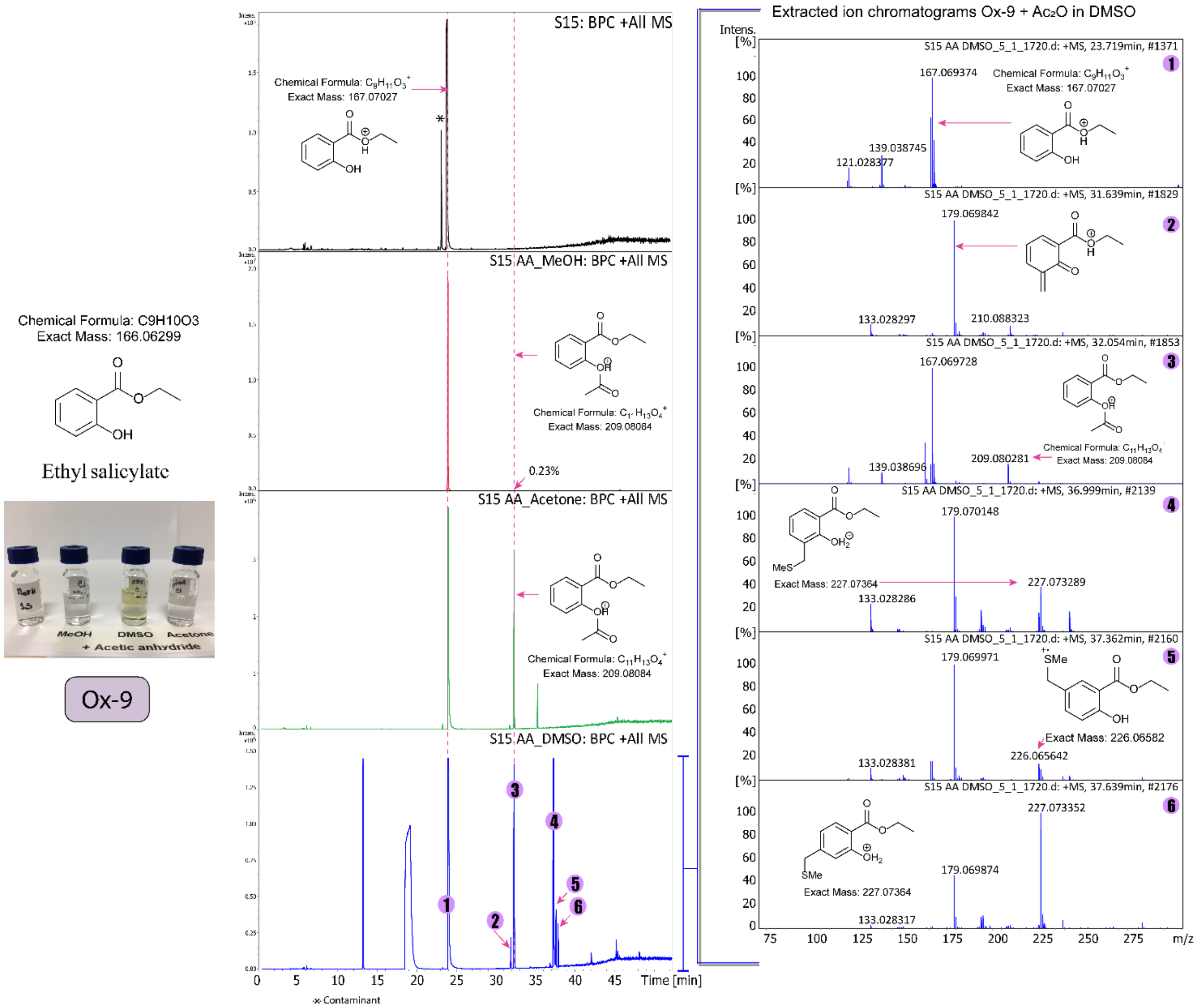

Fig. S9 Centre: chromatograms of Ox-9 without reaction (black), right: MS at different RT for the reaction in DMSO-AC$_2$O. A photo of the samples is embedded on the left-bottom of the figure. Suggested structures have been embedded in the figure.

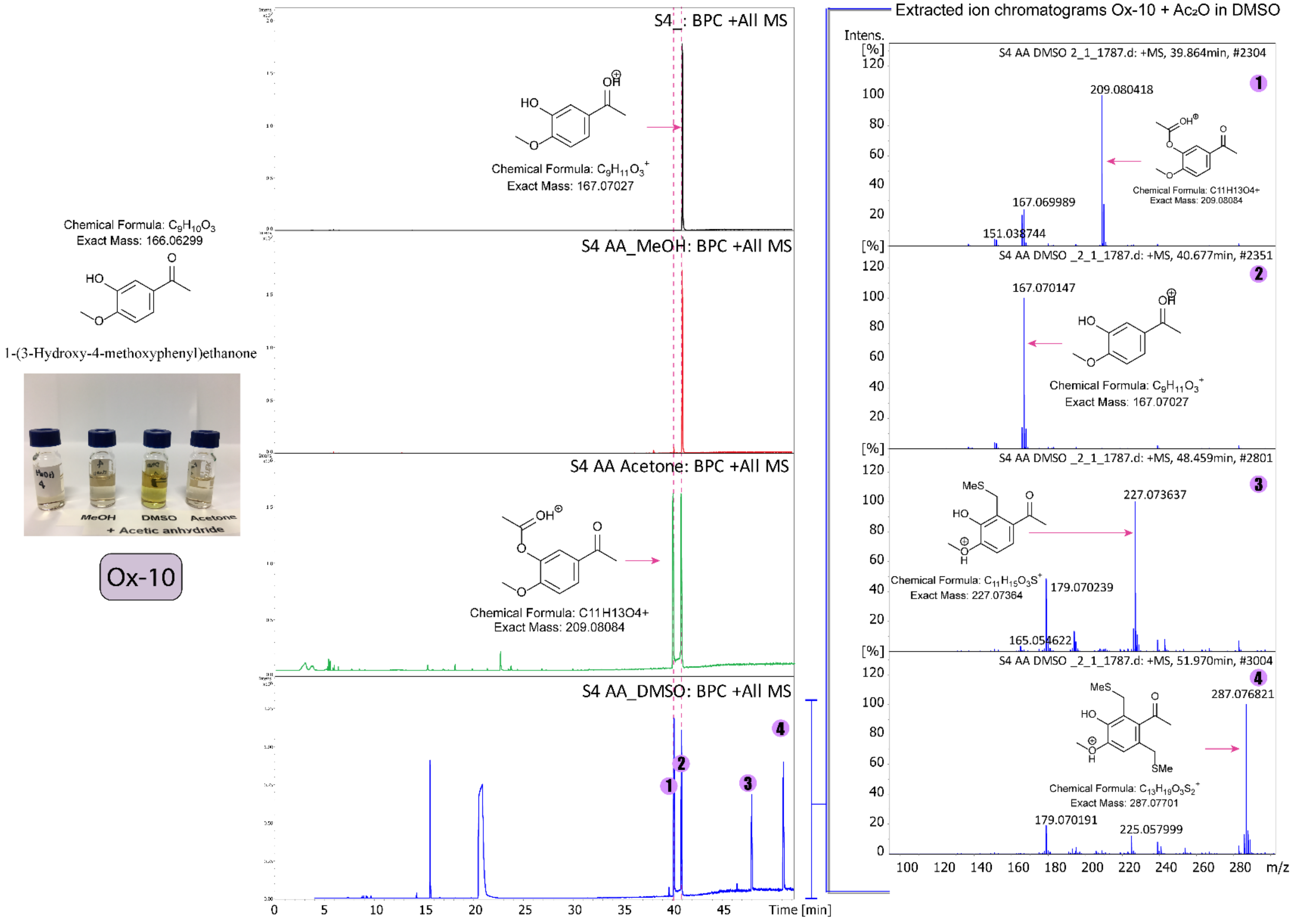

Fig. S10 Left: chromatograms of Ox-10 without reaction (black), right: MS at different RT for the reaction in DMSO-AC$_2$O. A photo of the samples is embedded on the left-bottom of the figure. Suggested structures have been embedded in the figure.

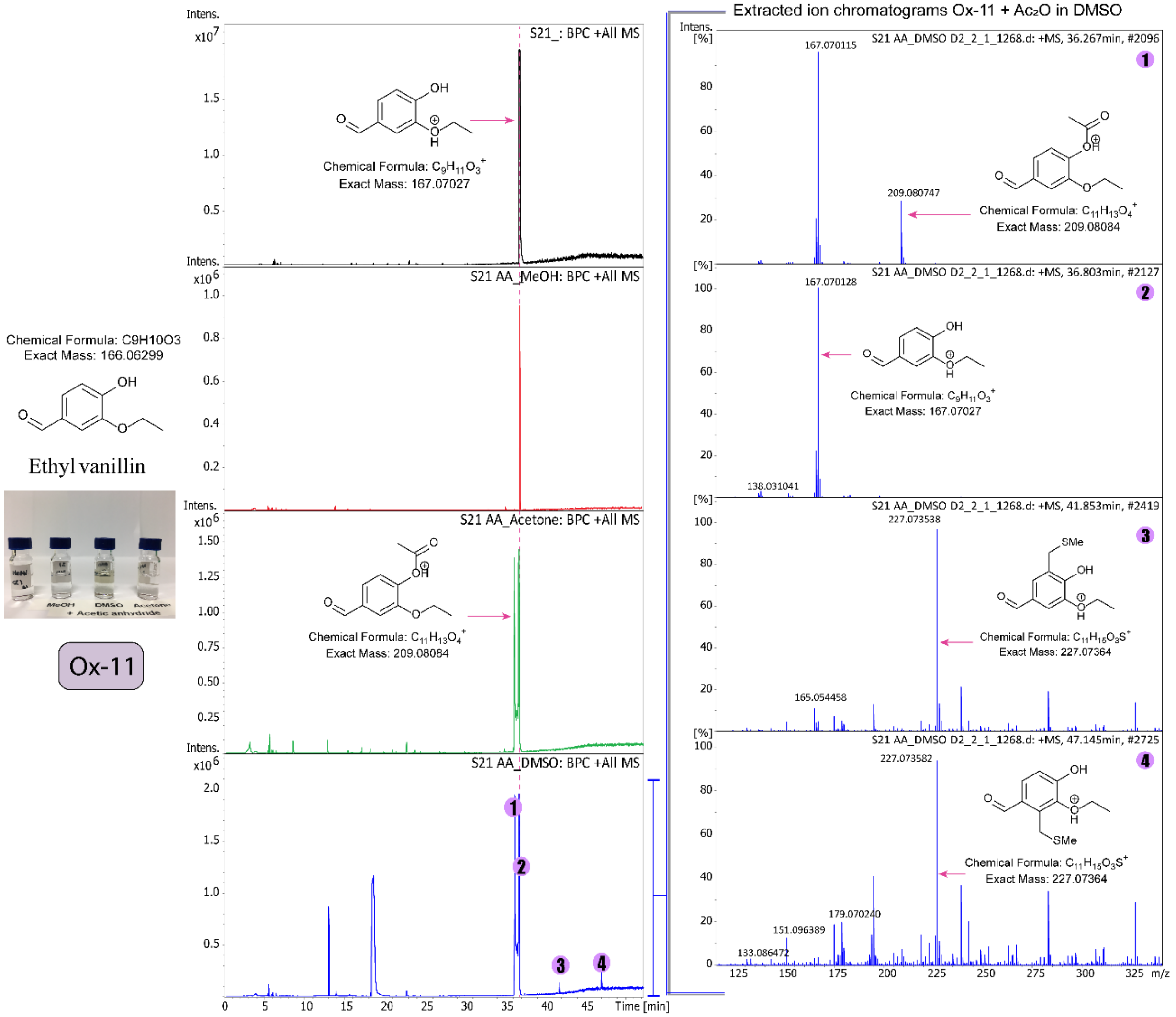

Fig. S11 Centre: chromatograms of Ox-11 without reaction (black), right: MS at different RT for the reaction in DMSO-AC$_2$O. A photo of the samples is embedded on the left-bottom of the figure. Suggested structures have been embedded in the figure.

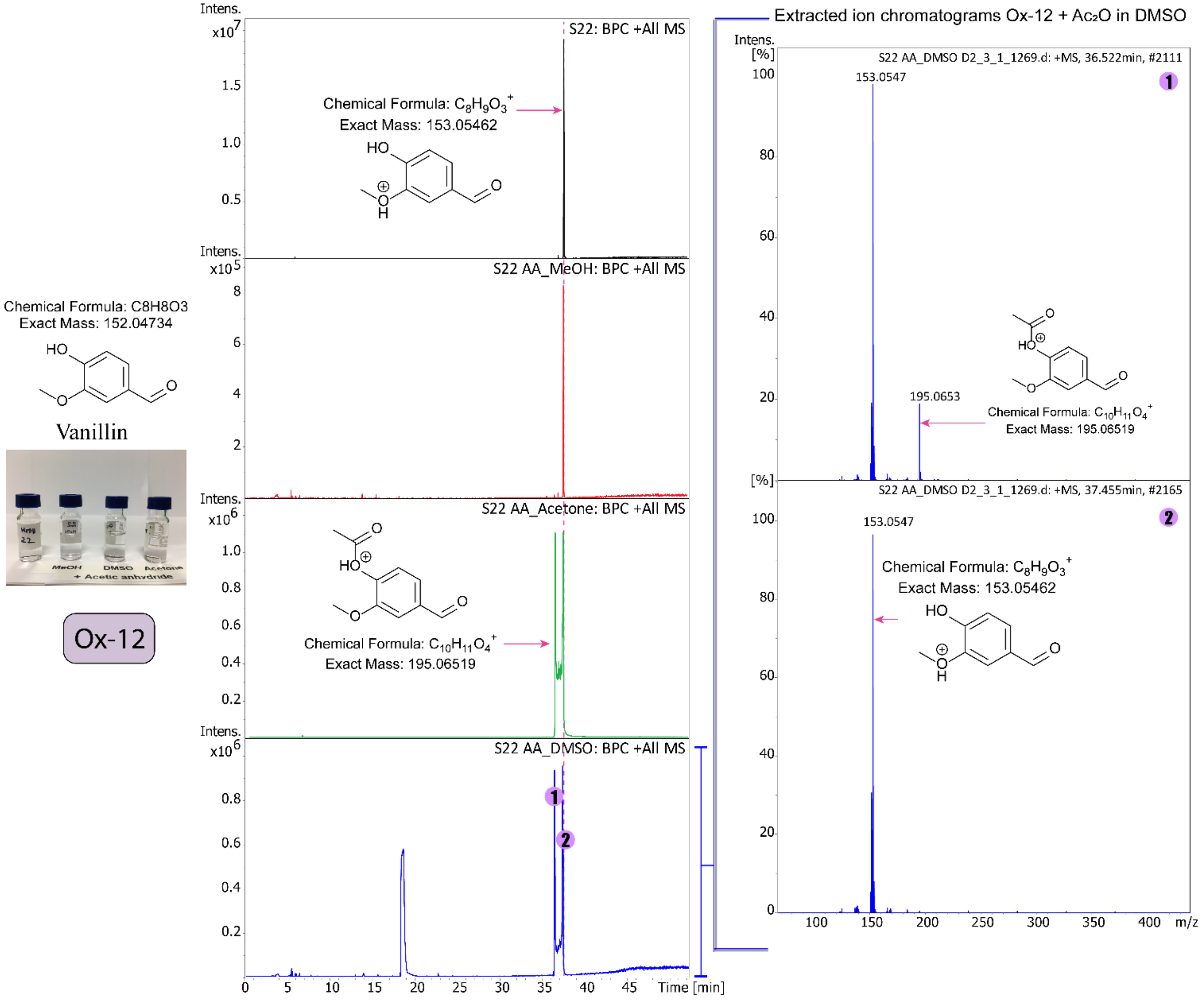

Fig. S12 Centre: chromatograms of Ox-12 without reaction (black), right: MS at different RT for the reaction in DMSO-AC$_2$O. A photo of the samples is embedded on the left-bottom of the figure. Suggested structures have been embedded in the figure.

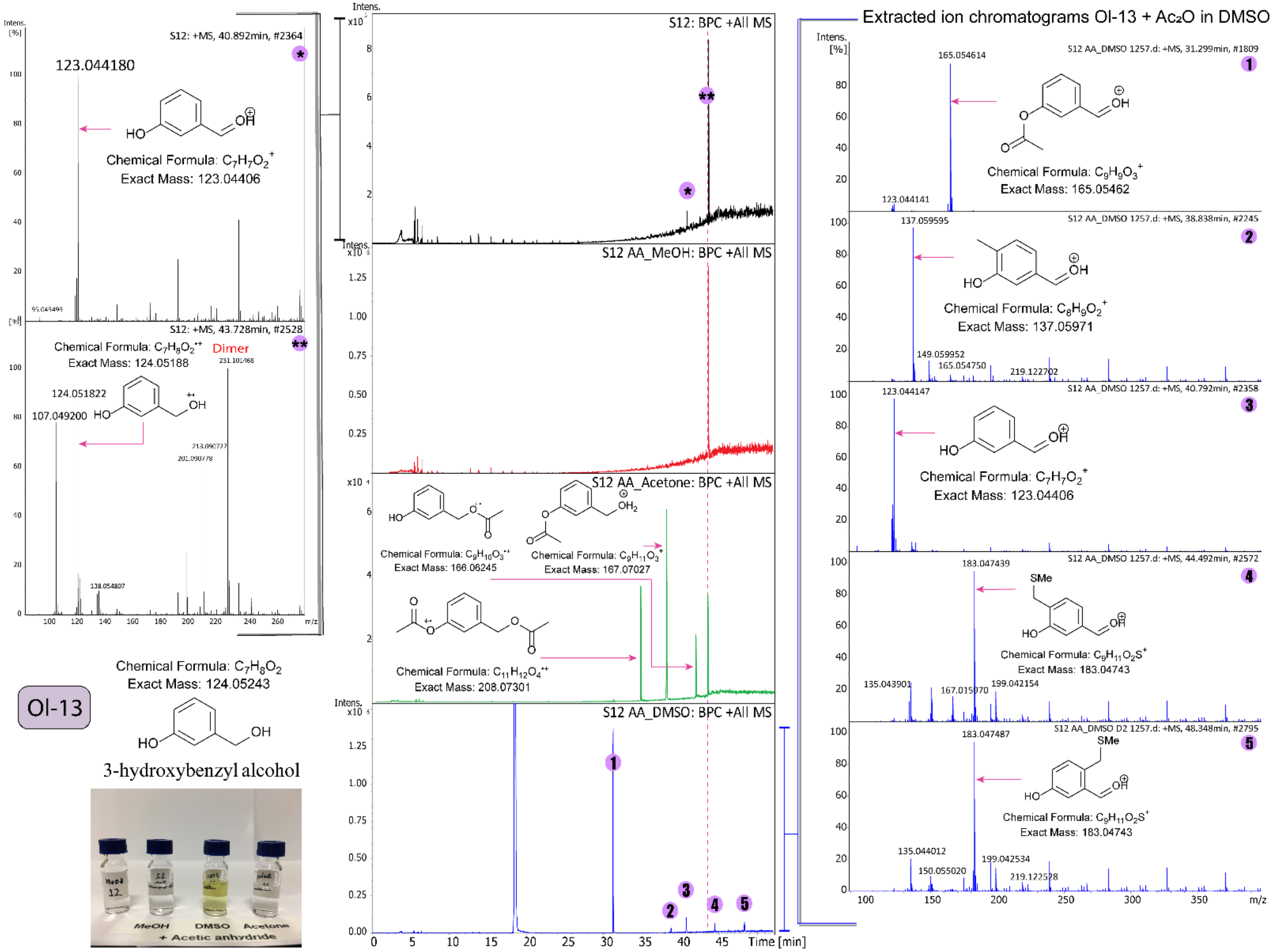

Fig. S13 Centre: chromatograms of OI-13 without reaction (black), left: extracted MS of raw standard, right: MS at different RT for the reaction in DMSO-AC₂O. A photo of the samples is embedded on the left-bottom of the figure. Suggested structures have been embedded in the figure.

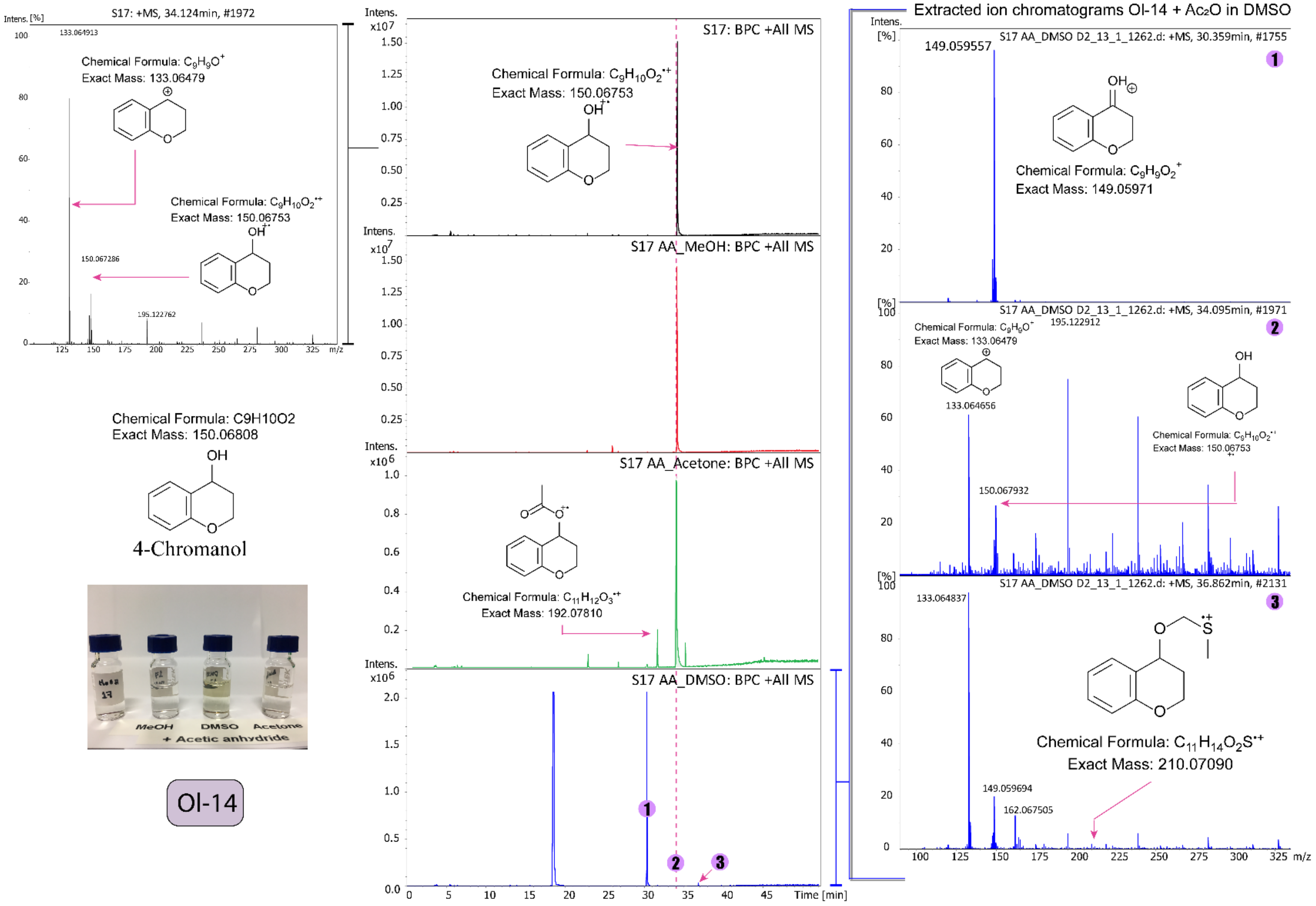

Fig. S14 Centre: chromatograms of OI-14 without reaction (black), left: extracted MS of raw standard, right: MS at different RT for the reaction in DMSO-AC₂O. A photo of the samples is embedded on the left-bottom of the figure. Suggested structures have been embedded in the figure.

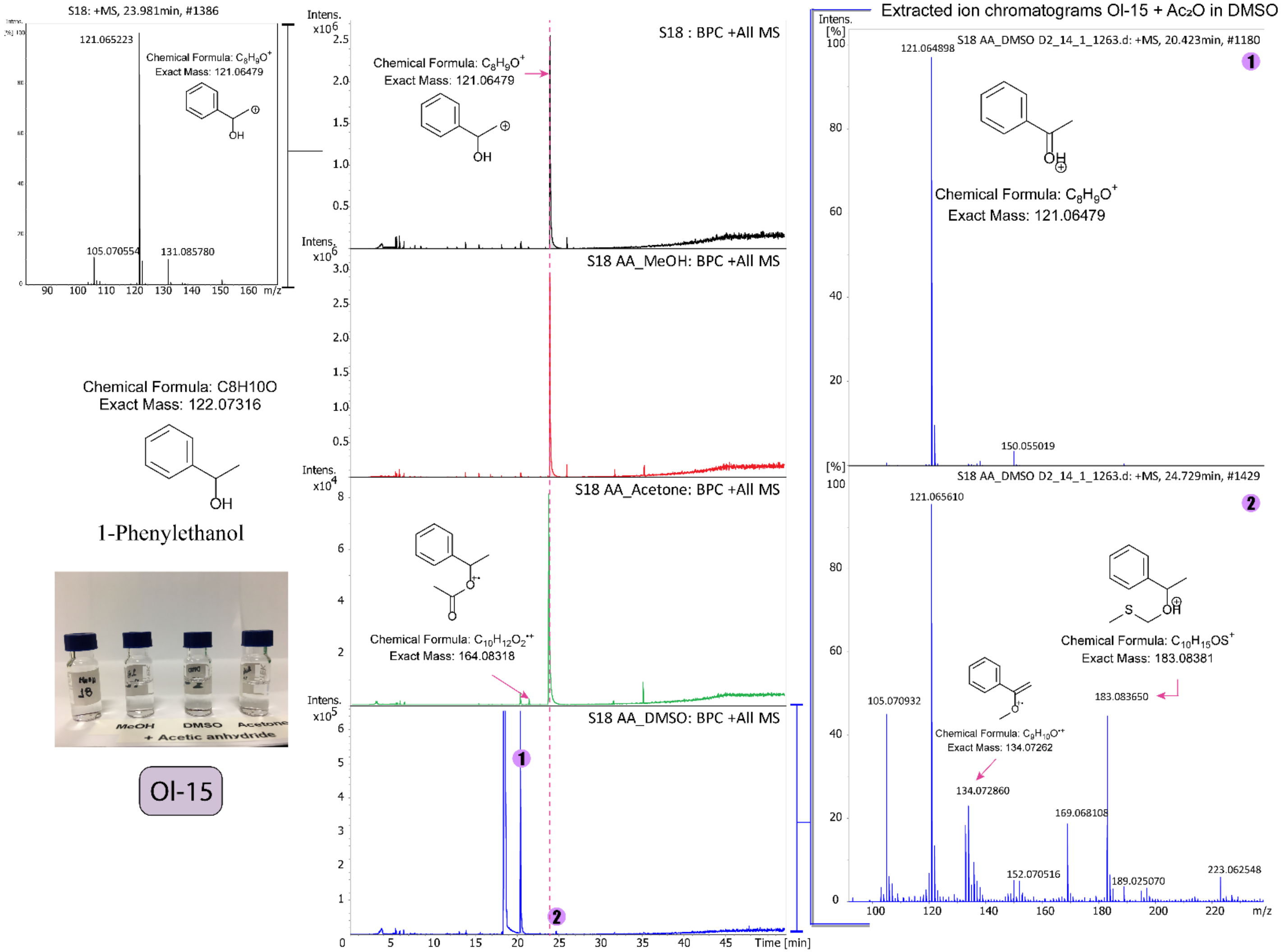

Fig. 15 Centre: chromatograms of Ol-15 without reaction (black), left: extracted MS of raw standard, right: MS at different RT for the reaction in DMSO-AC₂O. A photo of the samples is embedded on the left-bottom of the figure. Suggested structures have been embedded in the figure.

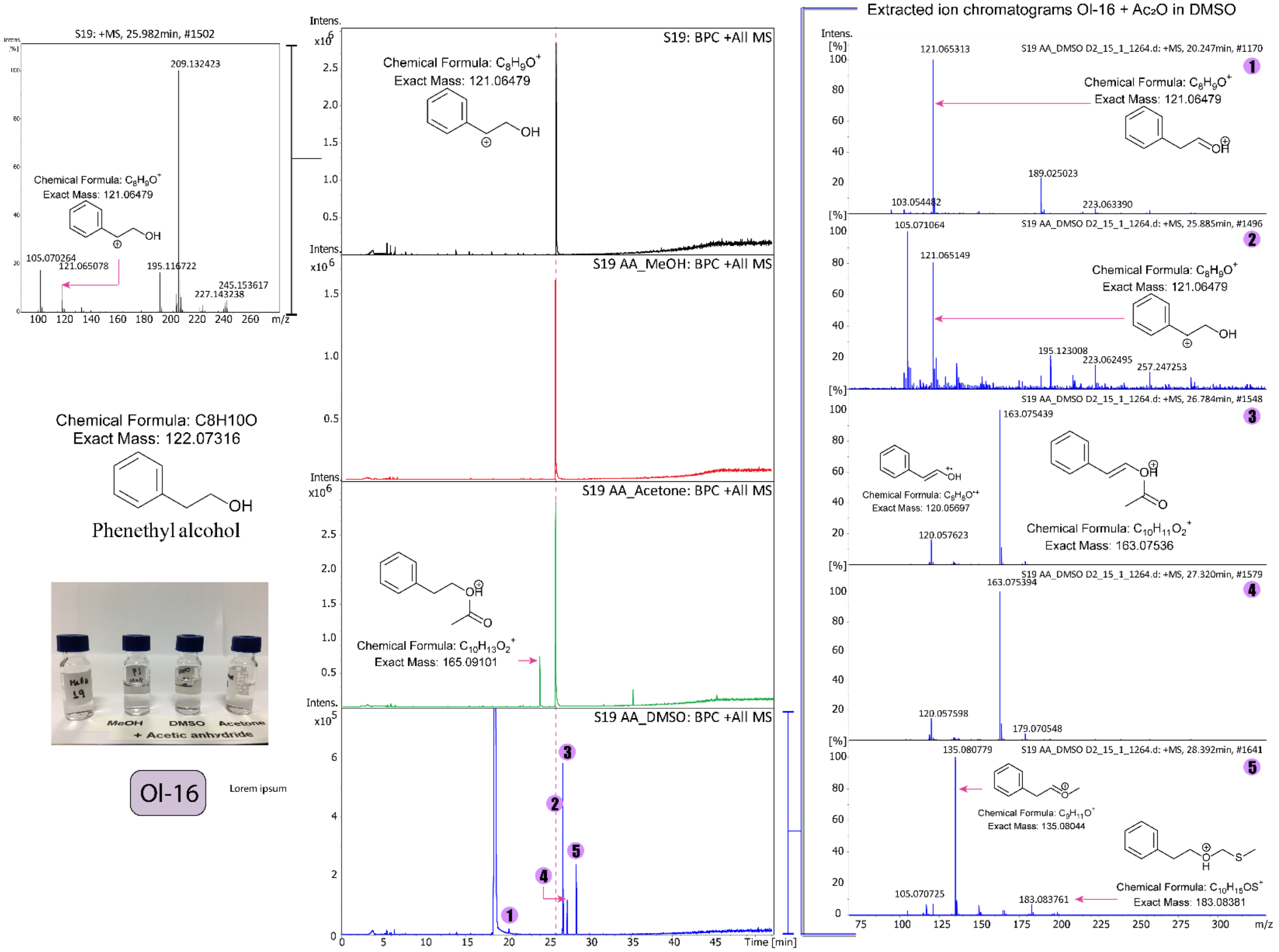

Fig. S16 Centre: chromatograms of OI-16 without reaction (black), left: extracted MS of raw standard, right: MS at different RT for the reaction in DMSO-AC₂O. A photo of the samples is embedded on the left-bottom of the figure. Suggested structures have been embedded in the figure.

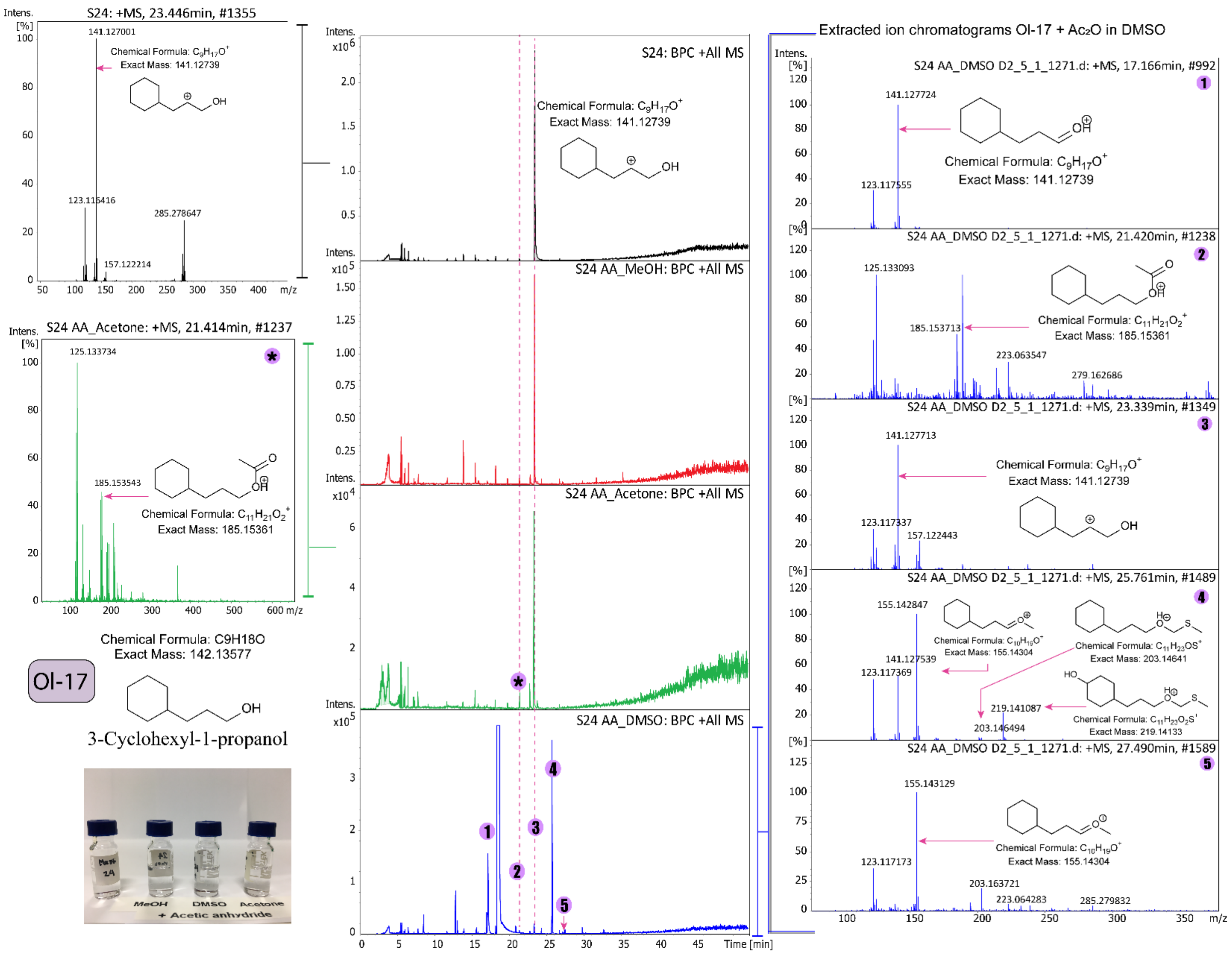

Fig. S17 Centre: chromatograms of Ol-17 without reaction (black), left: extracted MS of raw standard, right: MS at different RT for the reaction in DMSO-AC₂O. A photo of the samples is embedded on the left-bottom of the figure. Suggested structures have been embedded in the figure.

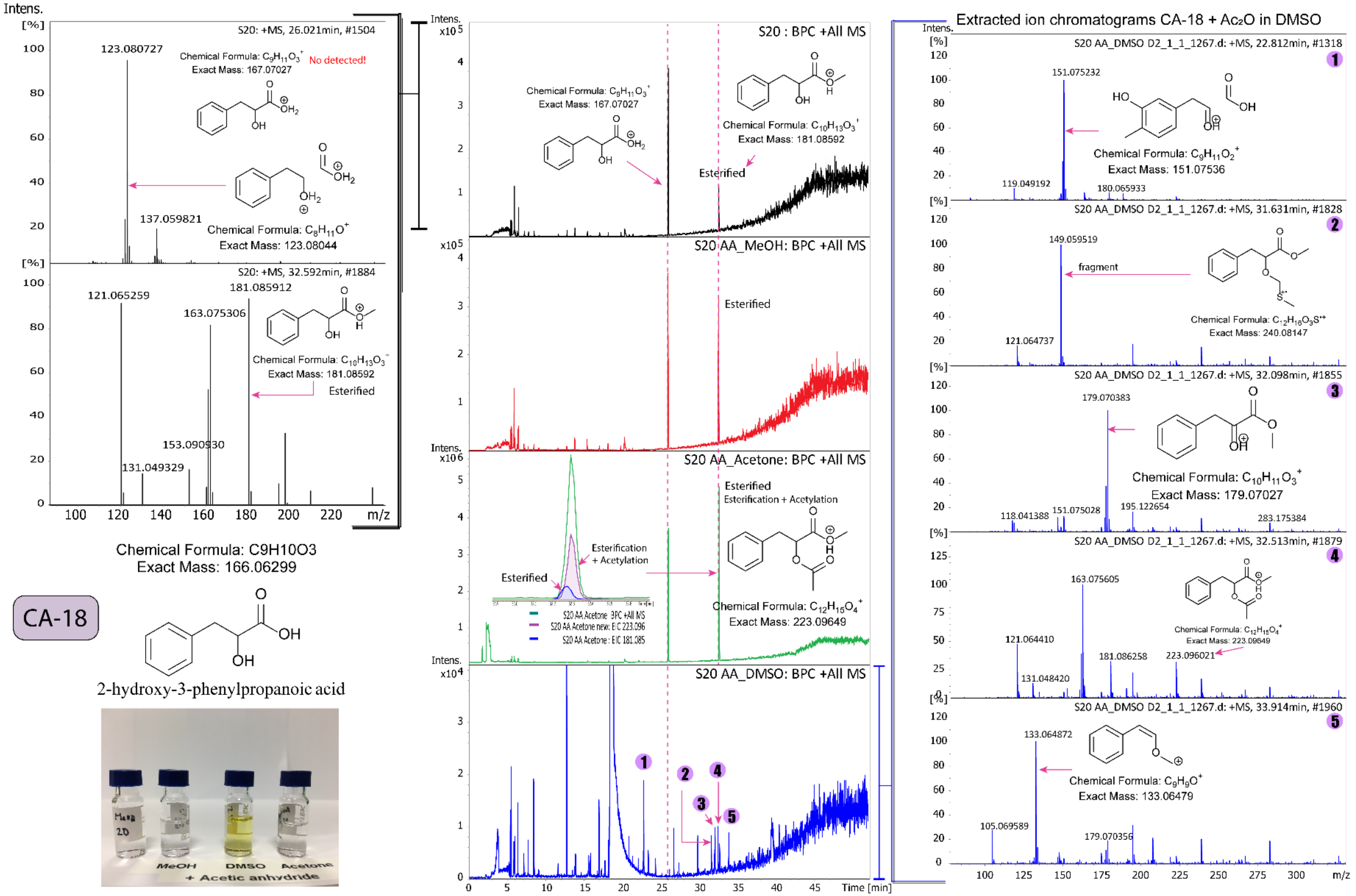

Fig. 18 Centre: chromatograms of CA-18 without reaction (black), left: extracted MS of raw standard, right: MS at different RT for the reaction in DMSO-AC₂O. A photo of the samples is embedded on the left-bottom of the figure. Suggested structures have been embedded in the figure.

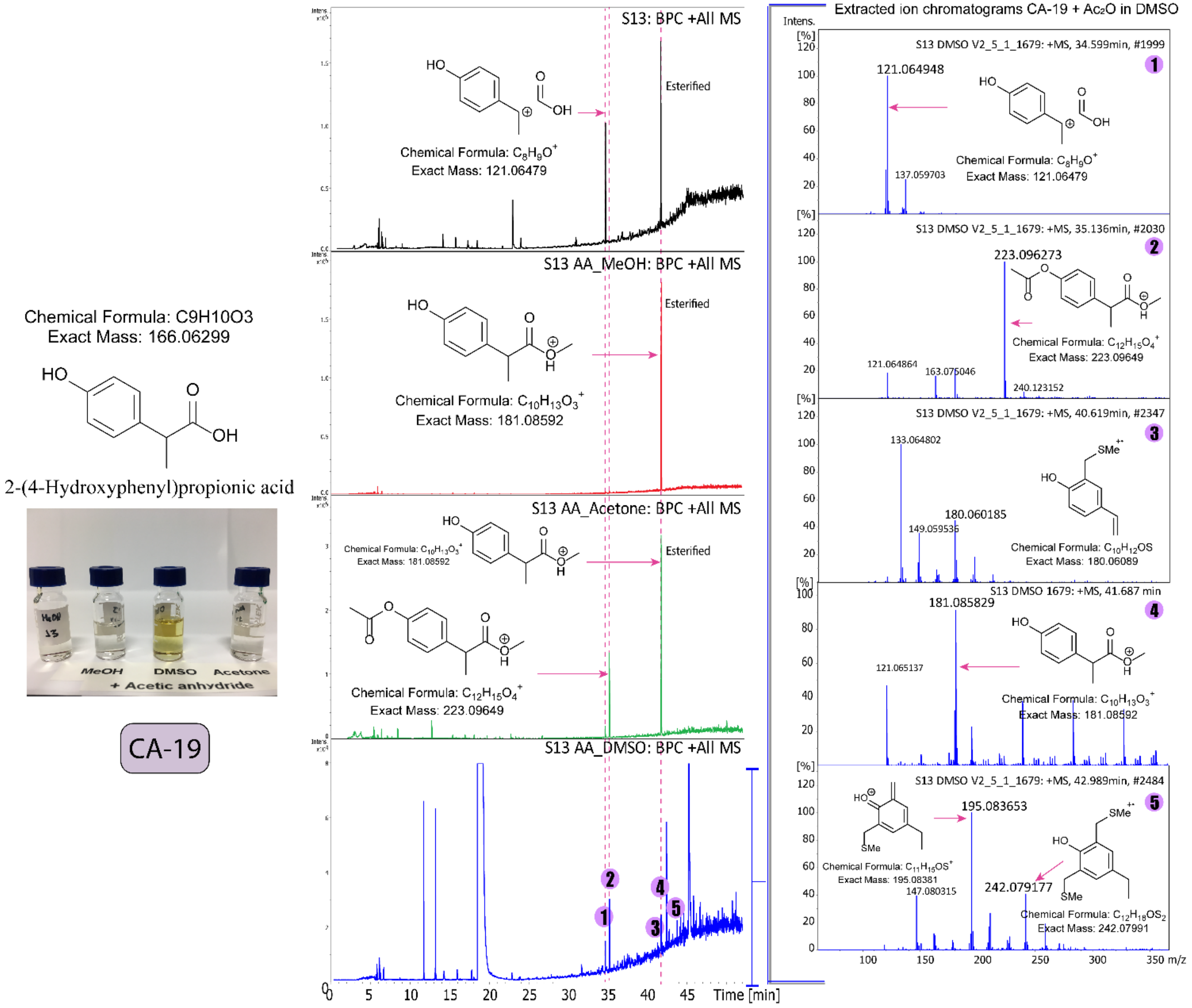

Fig. 19 Centre: chromatograms of CA-19 without reaction (black), right: MS at different RT for the reaction in DMSO-AC$_2$O. A photo of the samples is embedded on the left-bottom of the figure. Suggested structures have been embedded in the figure.

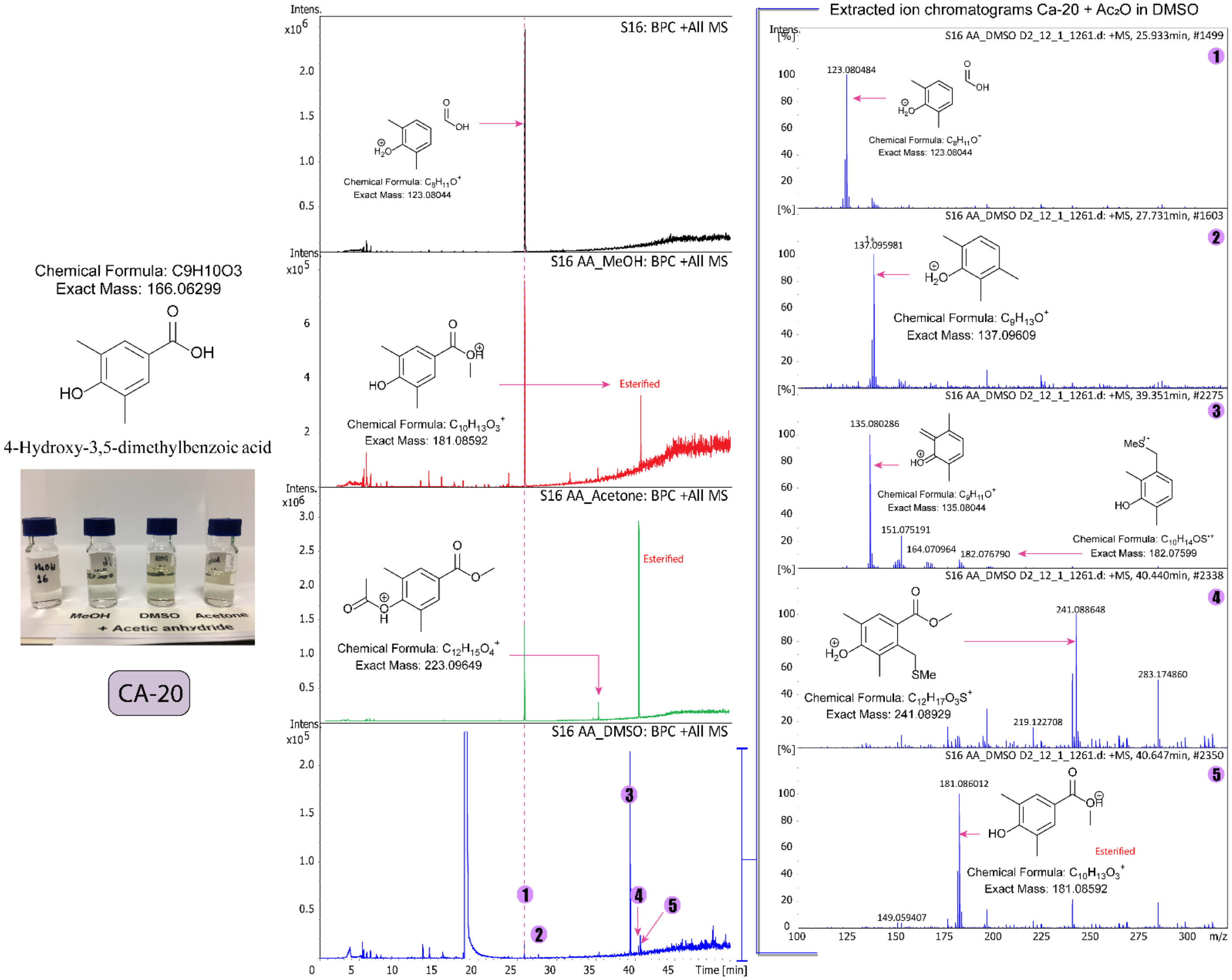

Fig. 20 Centre: chromatograms of CA-20 without reaction (black), right: MS at different RT for the reaction in DMSO-AC₂O. A photo of the samples is embedded on the left-bottom of the figure. Suggested structures have been embedded in the figure.

1.3 Reaction mechanisms

Mechanism of oxidation of alcohol to aldehyde:

The mechanism of the oxidation of alcohols to aldehydes by a combination of DMSO and acetic anhydride is a known reaction.[1] The mechanism for this reaction is illustrated below.

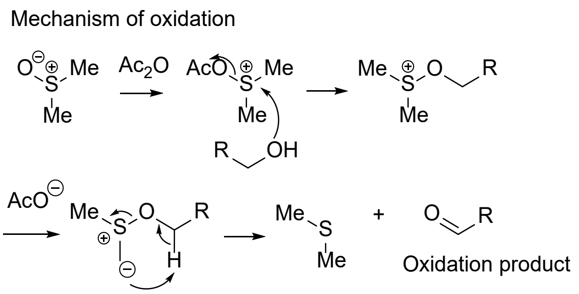

The mechanism of formation of MTM derivatives of the phenols is shown below – this involves formation of a cationic intermediate followed by its reaction with the phenols:

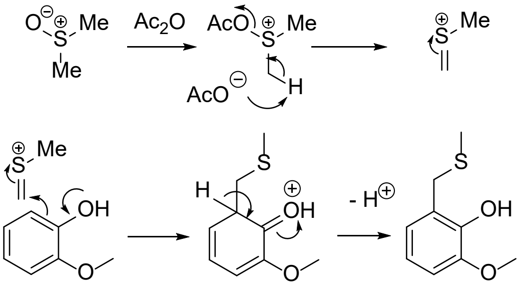

Loss of SMe (possibly acid promoted) leads to a cation, which is also observed:

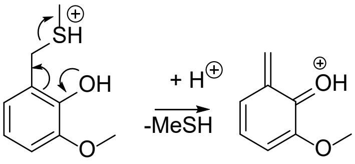

Aldehydes are formed from some alcohols in their reactions with DMSO/acetic anhydride, and these have the potential to undergo further reactions with organic molecules under the reaction conditions. These reactions would take place through an aldol condensation in which a new C-C bond is formed between the C of the C=O bond of the aldehyde and the alpha-C (relative to a C=O) of a ketone. This may be followed by elimination to an alkene. A phenol could also potentially react with an aldehyde under the reaction conditions to give an addition product. However, there was no evidence of products of this type in our study.

(1) J. D. Albright and L. Goldman, J. Am. Chem. Soc., 1967, 89, 2416–2423.

1.4 Reactions

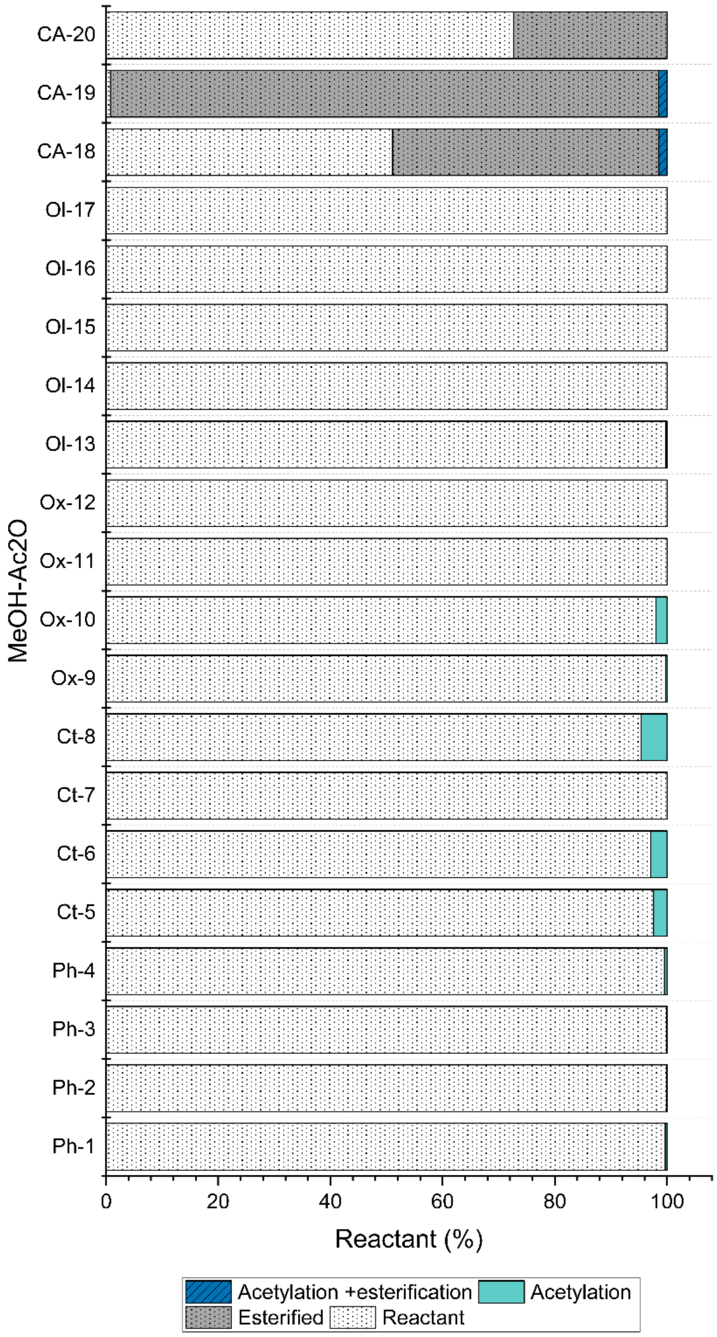

Fig. S21 Reaction products observed in MeOH-Ac$_2$O mixtures.

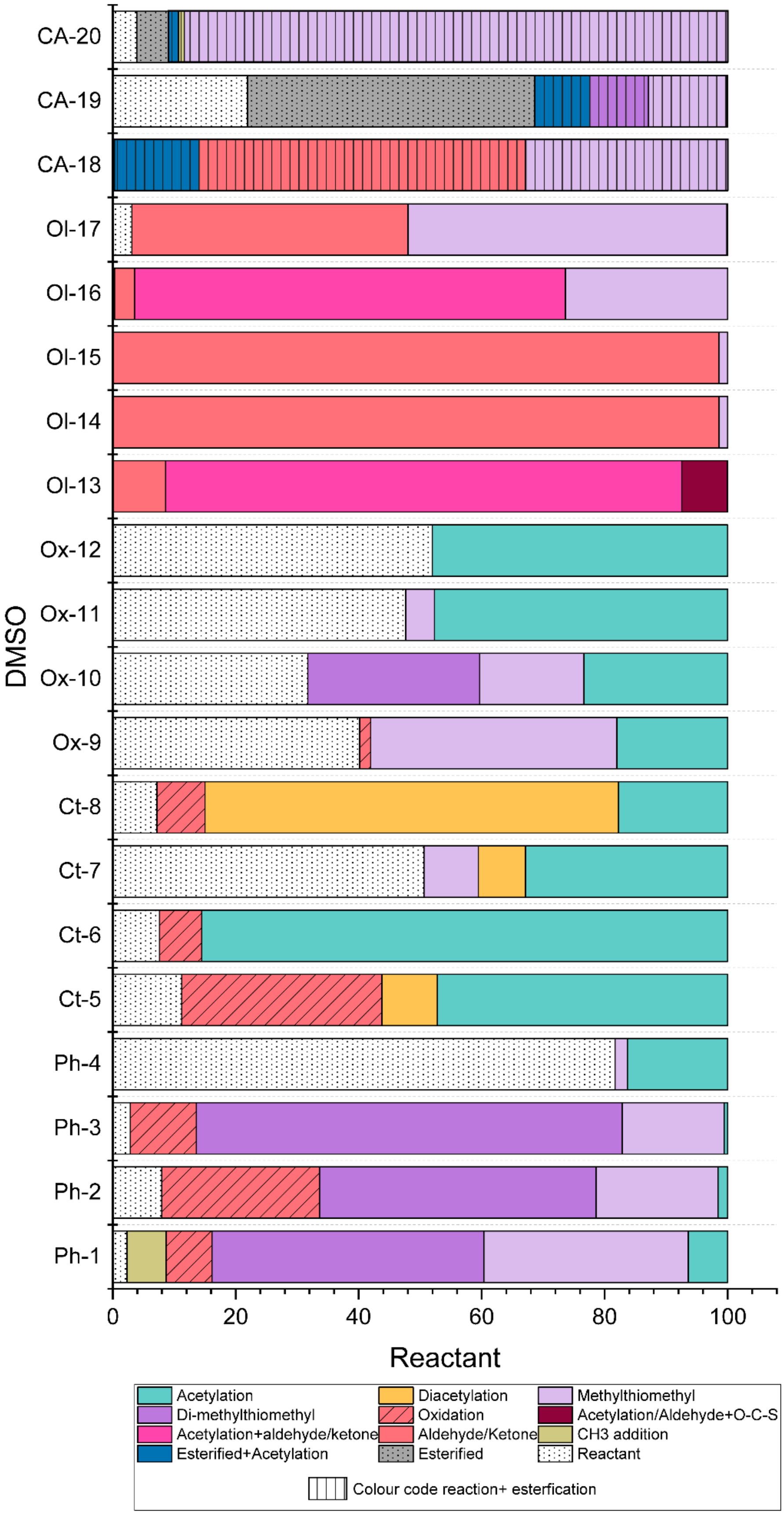

Fig. S22 Detailed reactions observed in DMSO-AC$_2$O mixtures.

1.5 Mass spectra

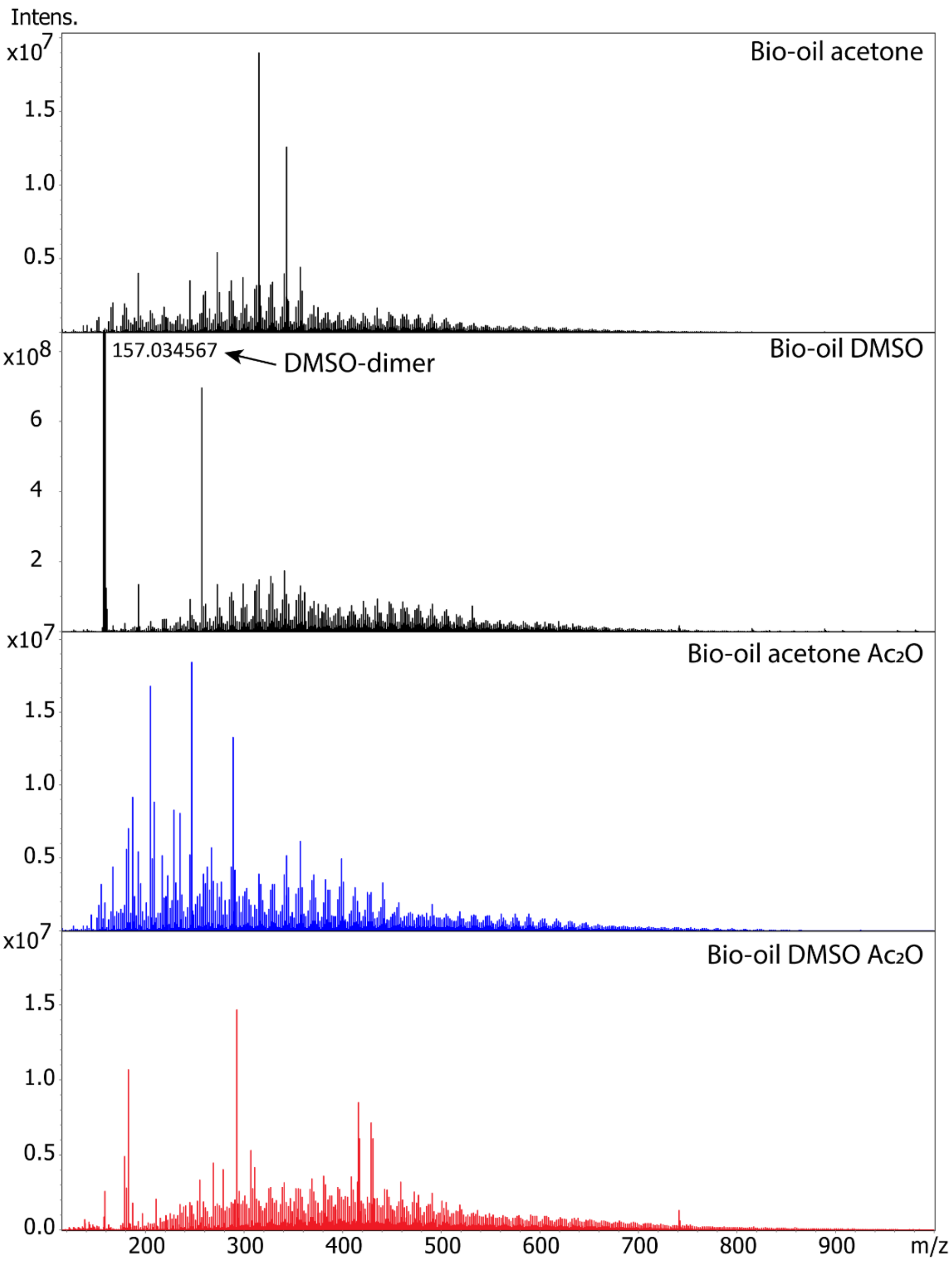

Fig. S23 Mass spectra of the bio-oil before and after derivatisation.

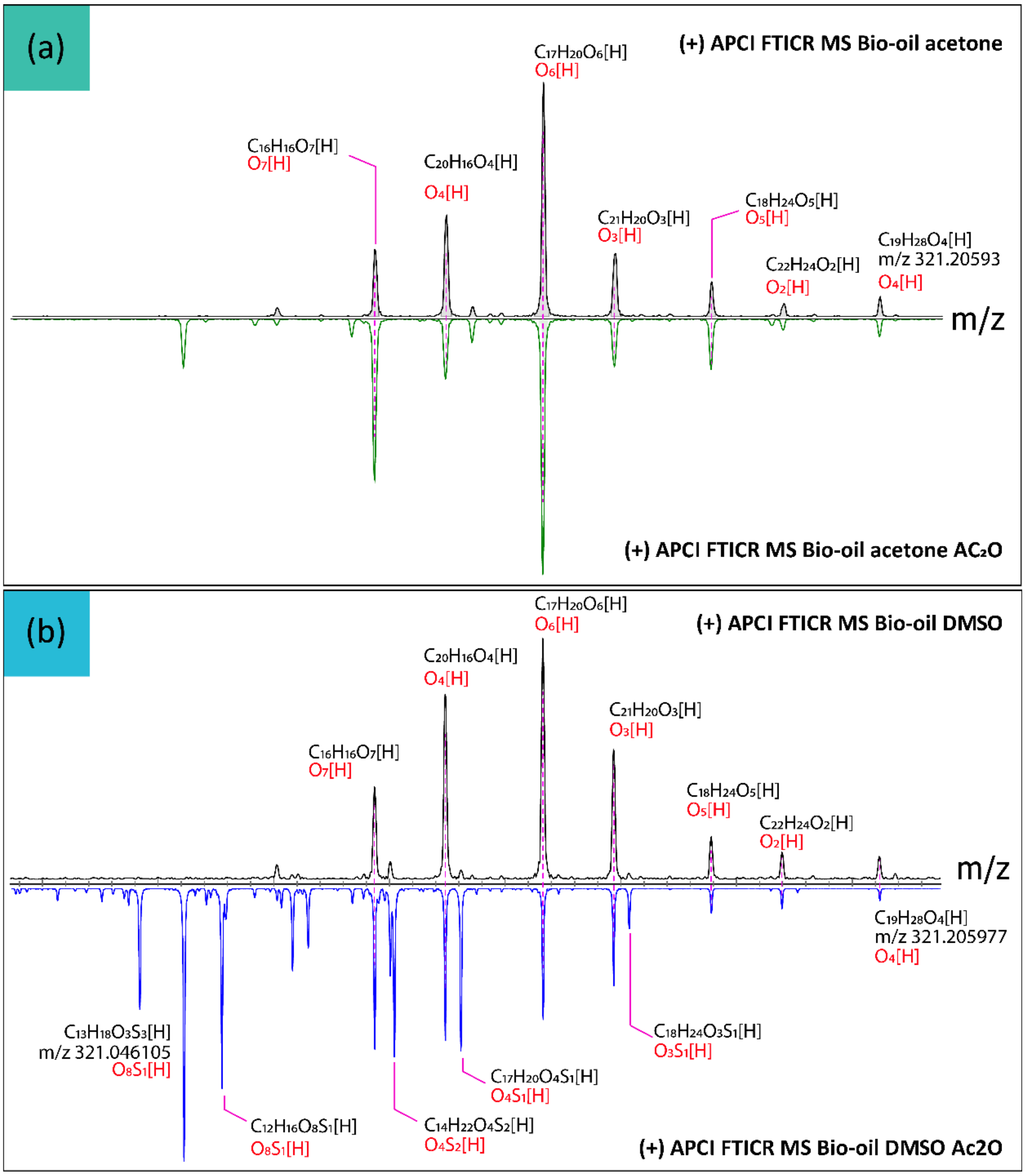

Fig. S24 Zoomed-in mass spectra at m/z 321. (a) bio-oil in acetone and (b) bio-oil in DMSO. Mixtures dopped with Ac_2O have been inverted in the y-axis.

1.6 Van Krevelen and DBE plots

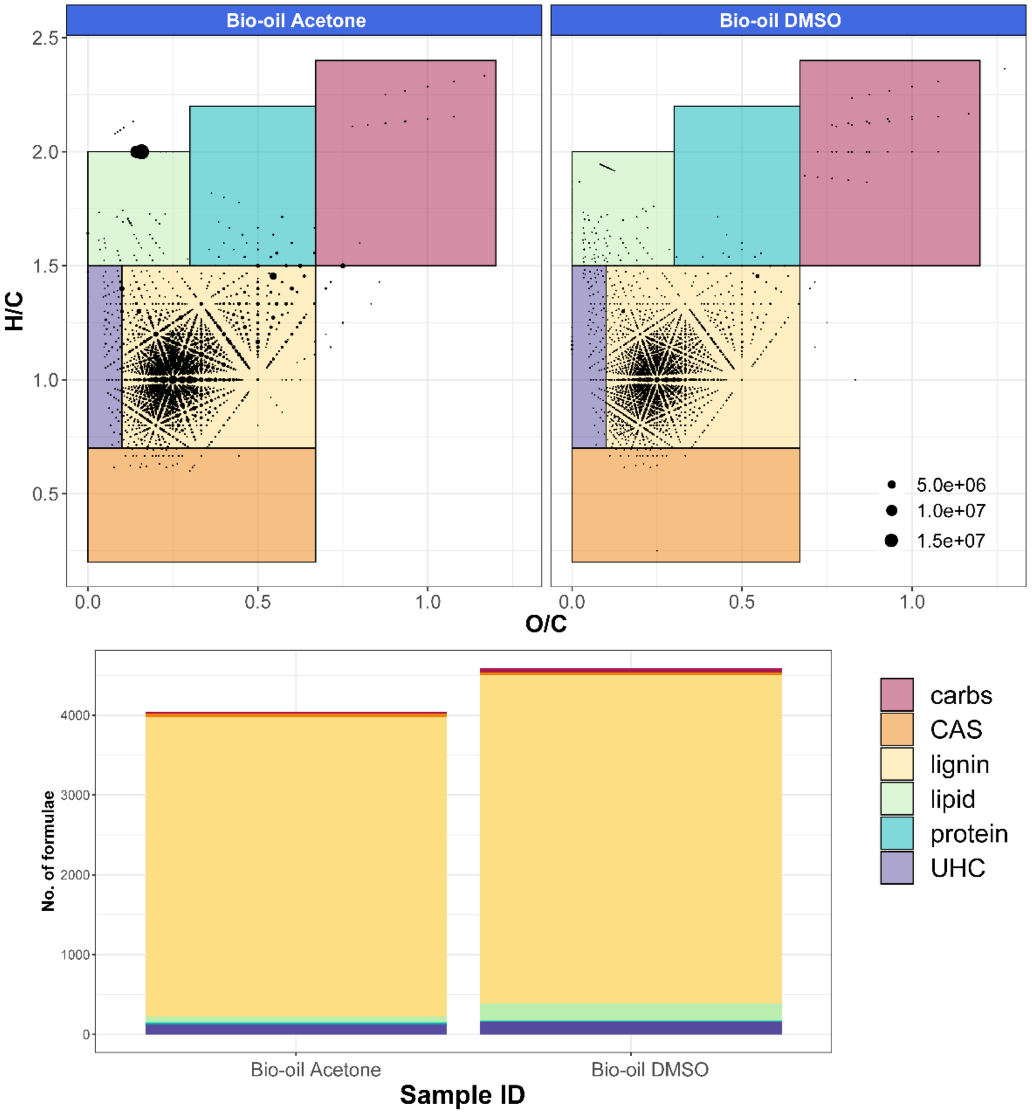

Fig. S25 Van Krevelen diagram for the bio-oil samples blanks (acetic anhydride not added to the mixture). Bottom, relative contribution by category Carbs: carbohydrates, CAS: condensed aromatic ring structures and UHC: unsaturated hydrocarbons

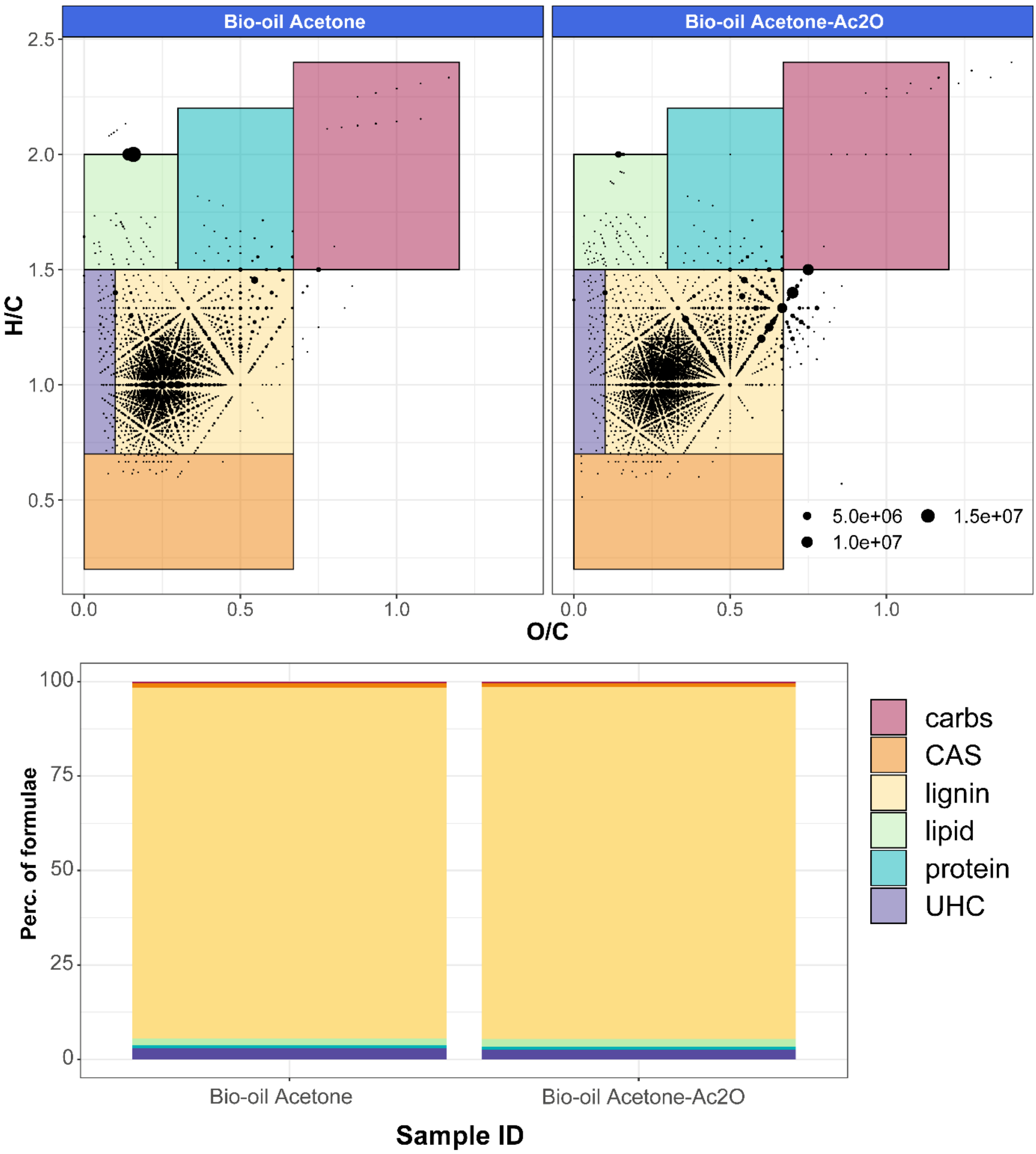

Fig. S26 Van Krevelen diagram for the bio-oil samples in acetone. Bottom, relative contribution by category Carbs: carbohydrates, CAS: condensed aromatic ring structures and UHC: unsaturated hydrocarbons.

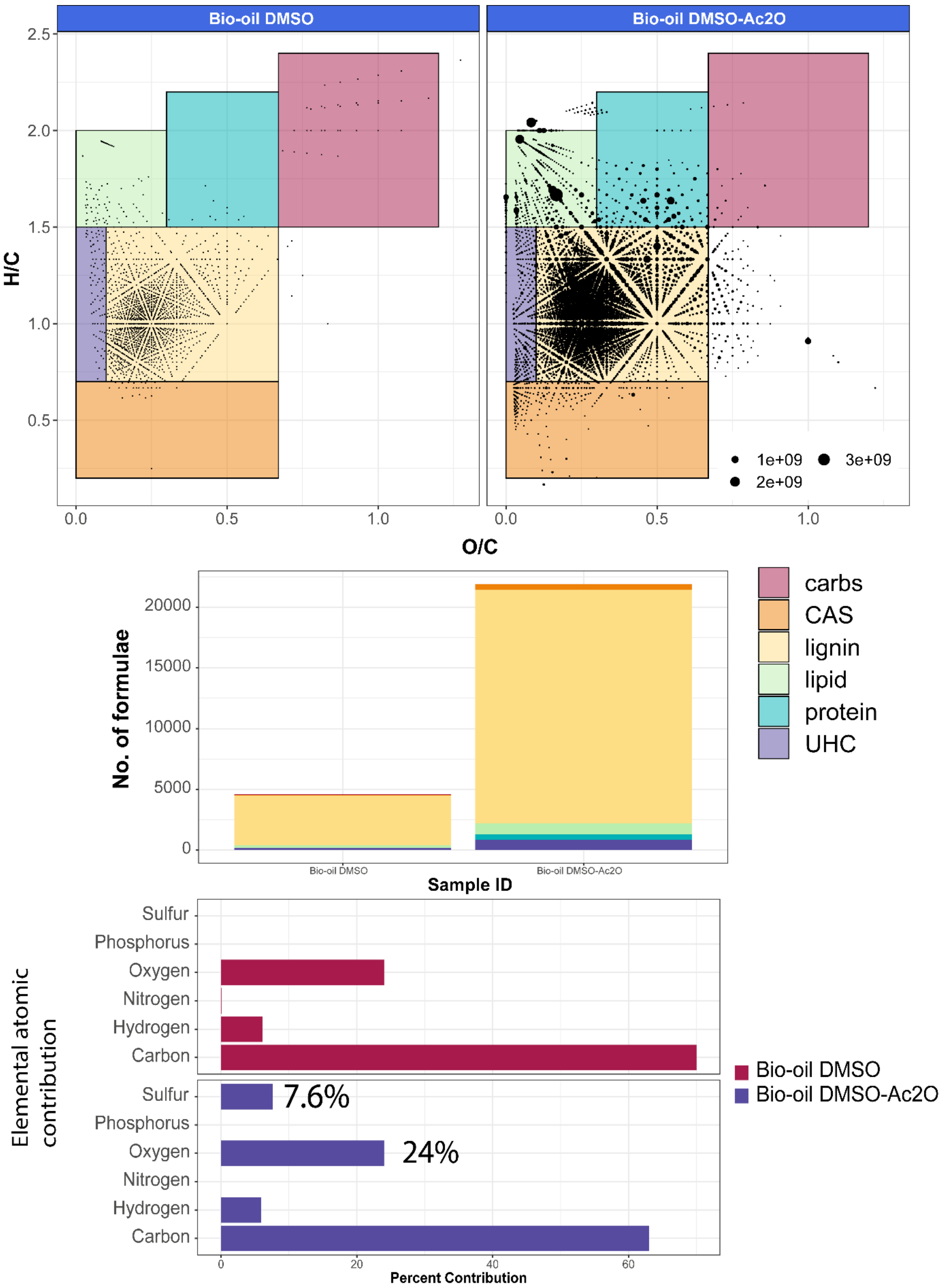

Fig. S27 Top: van Krevelen diagram for the bio-oil samples in DMSO. Centre, number of compositions by category; Carbs: carbohydrates, CAS: condensed aromatic ring structures and UHC: unsaturated hydrocarbons. Bottom: atomic contribution calculated using the detected molecular compositions.

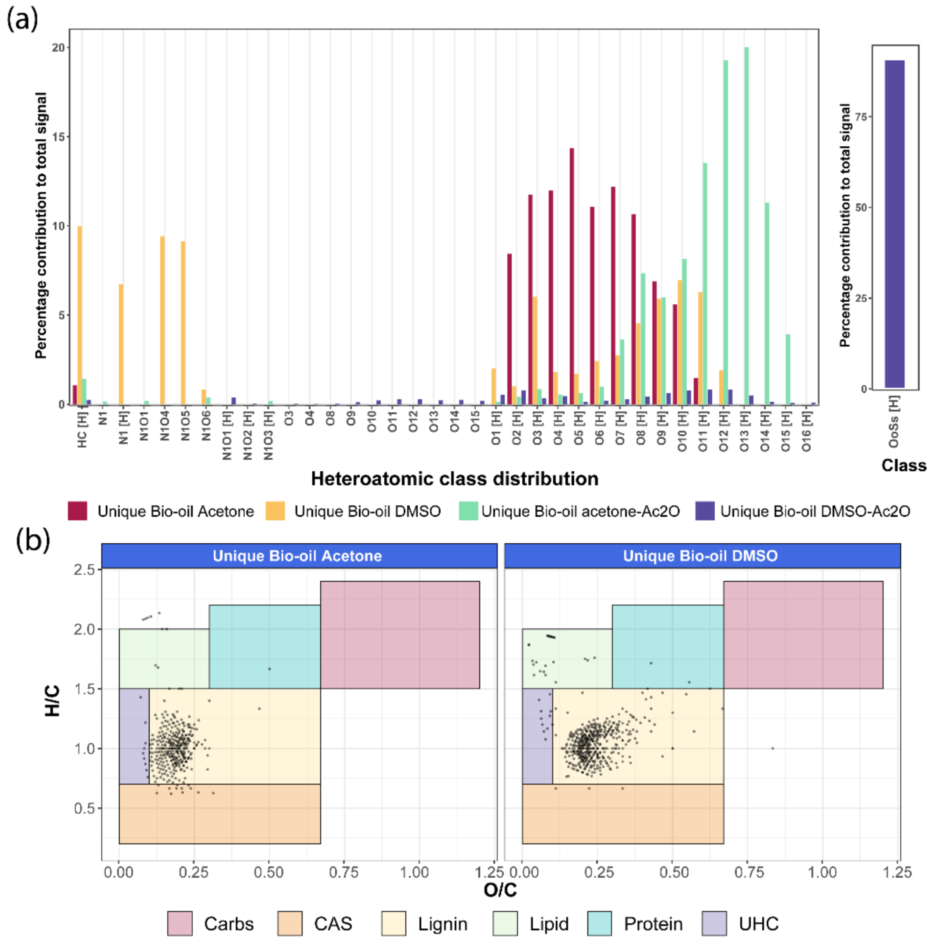

Fig. S28 (a) class distribution of unique elemental compositions weighted by relative contribution to the total intensity. (b) van Krevelen plot of the unique molecular compositions detected in the blanks. Coloured boxes are used to indicate compositions classified as carbohydrates (carbs), CAS: condensed aromatic ring structures, UHC: unsaturated hydrocarbons, lignin, lipids and proteins.

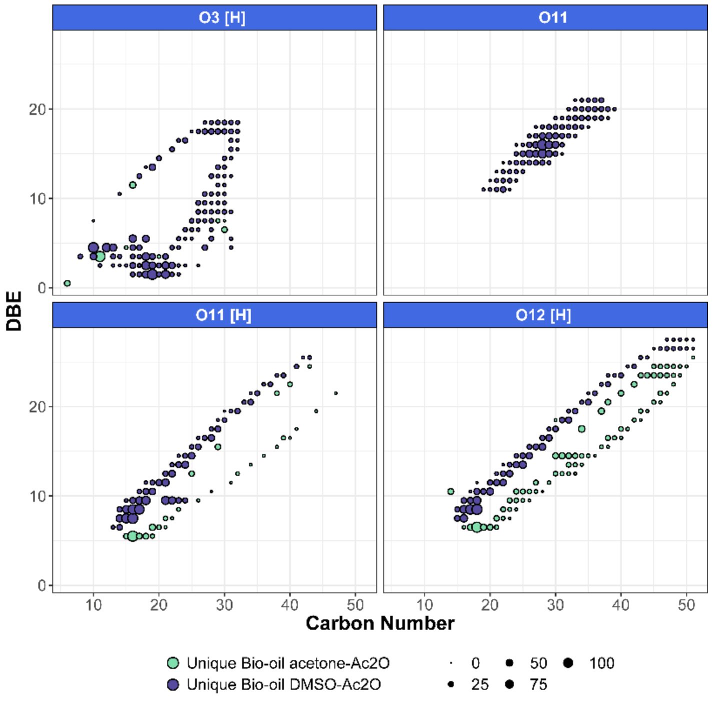

Fig. S29 Double bond equivalent plots of the unique compositions detected in Acetone-Ac$_2$O and DMSO-Ac$_2$O mixtures.

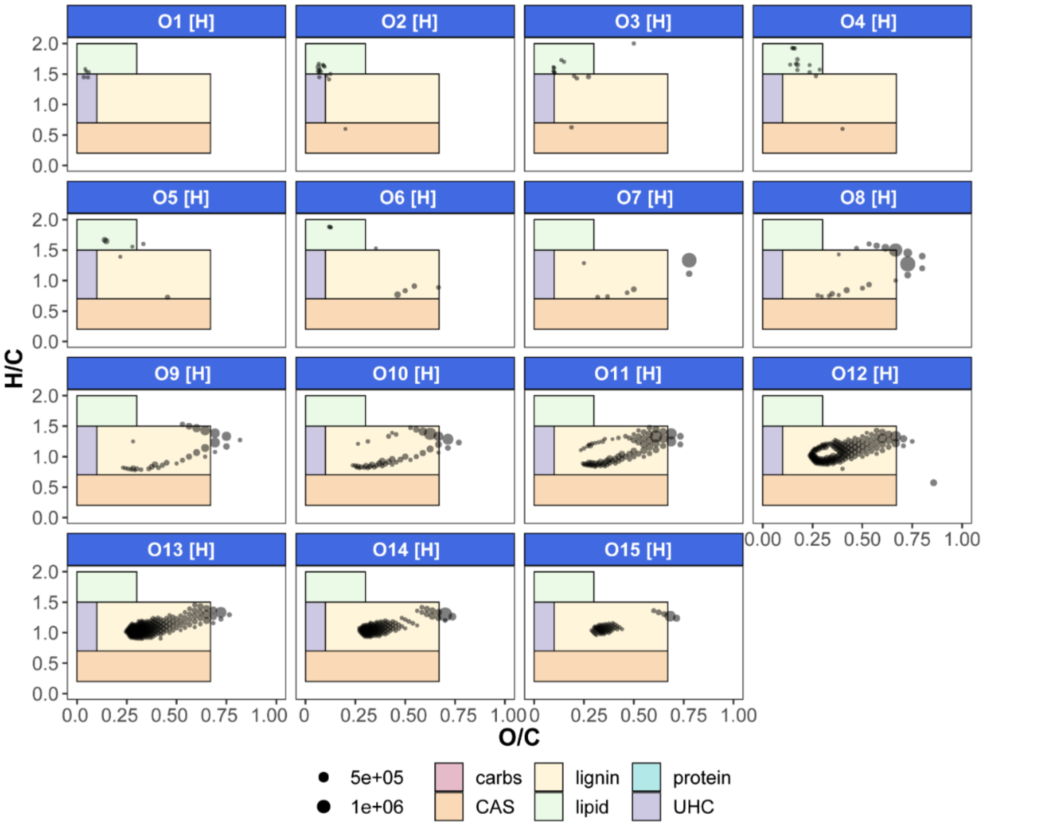

Fig. S30 Van Krevelen diagrams of even-electron ion species detected in the unique in Bio-oil acetone-Ac$_2$O.

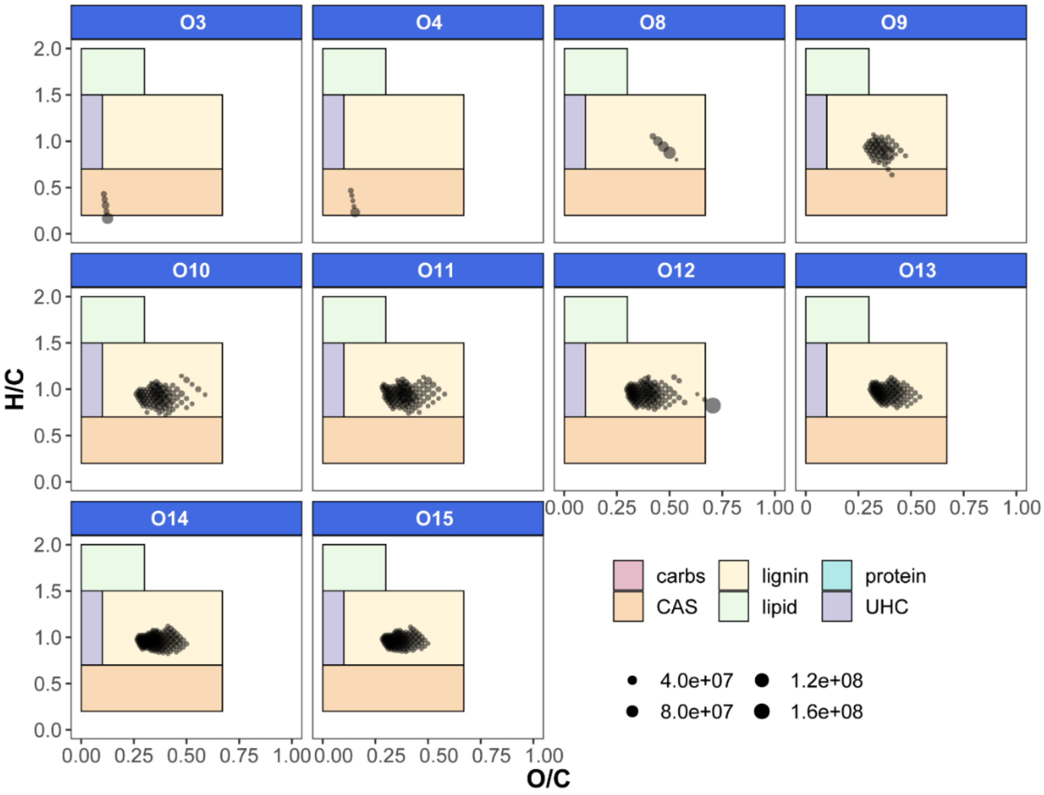

Fig. S31 Van Krevelen diagrams of odd-electron ion species detected in the unique in Bio-oil DMSO-Ac$_2$O.

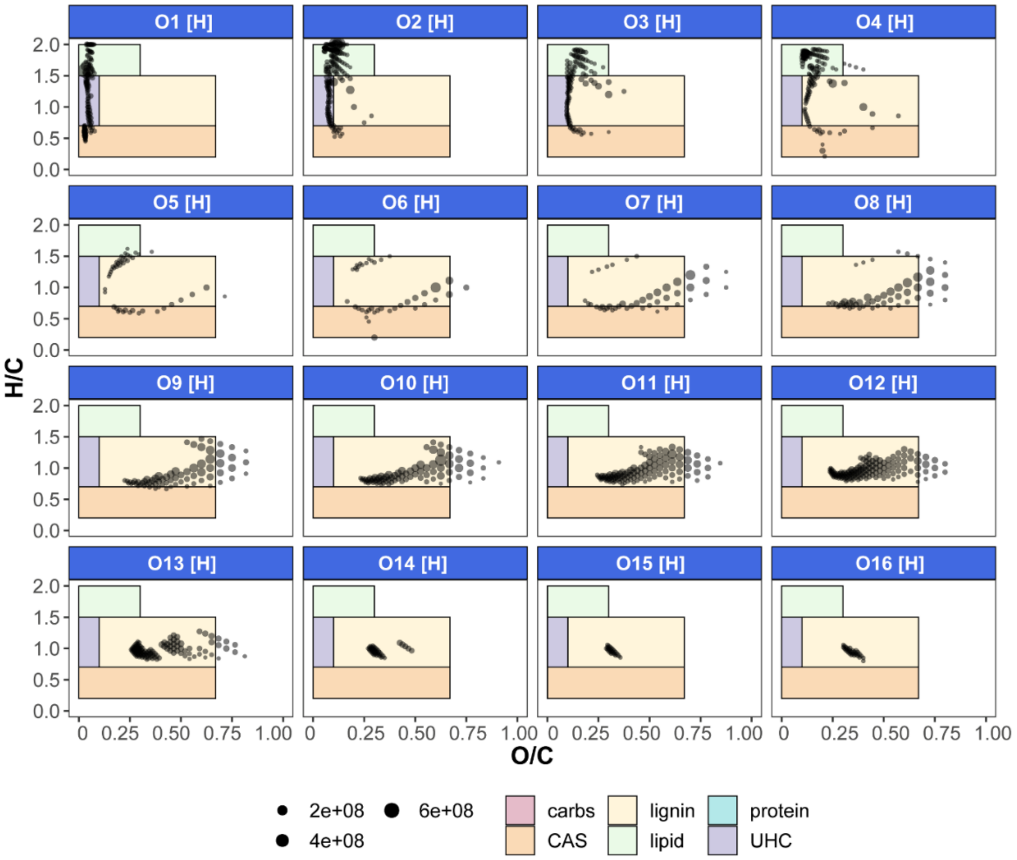

Fig. S32 Van Krevelen diagrams of even-electron ion species detected in the unique in Bio-oil DMSO-Ac$_2$O.

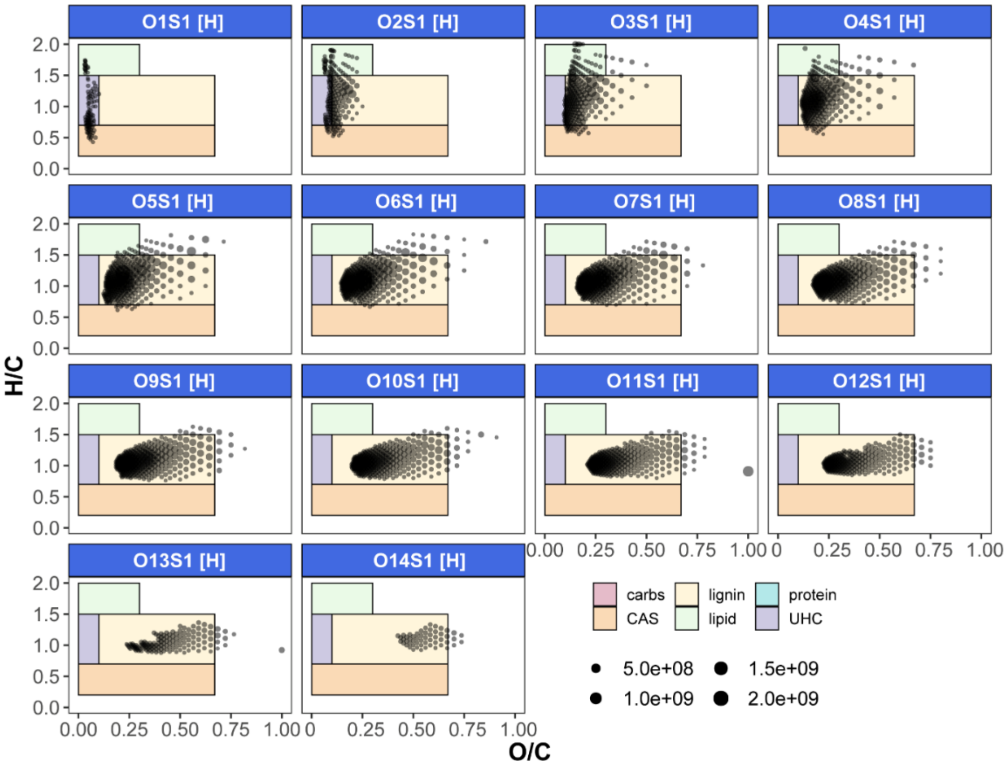

Fig. S33 Van Krevelen diagrams of O$_O$S$_1$[H] species detected in the unique in Bio-oil DMSO-Ac$_2$O.

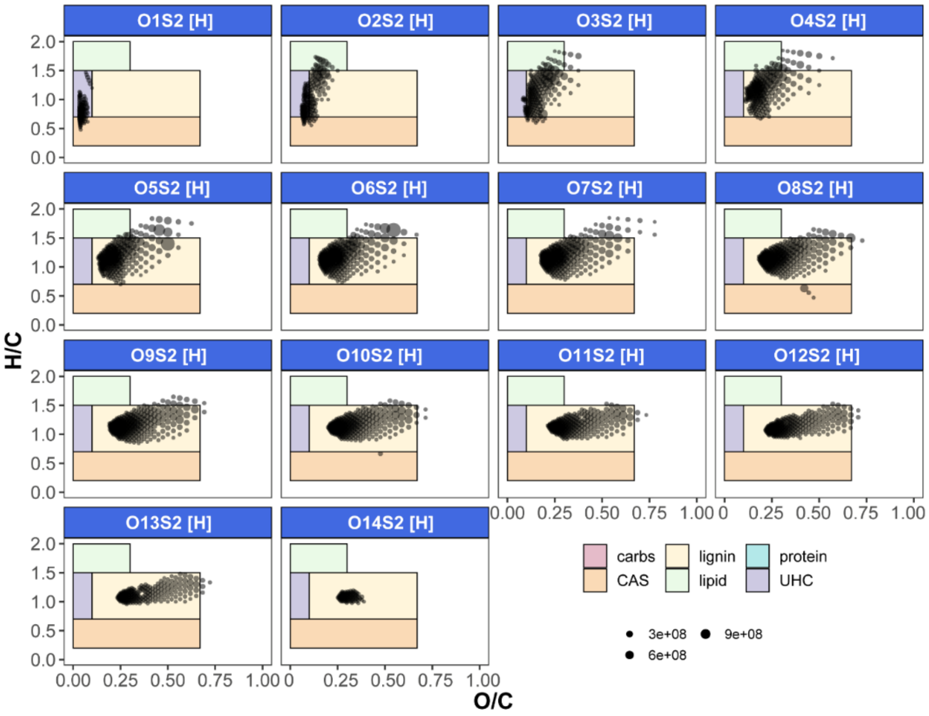

Fig. S34 Van Krevelen diagrams of O_OS_2[H] species detected in the unique in Bio-oil DMSO-Ac_2O.

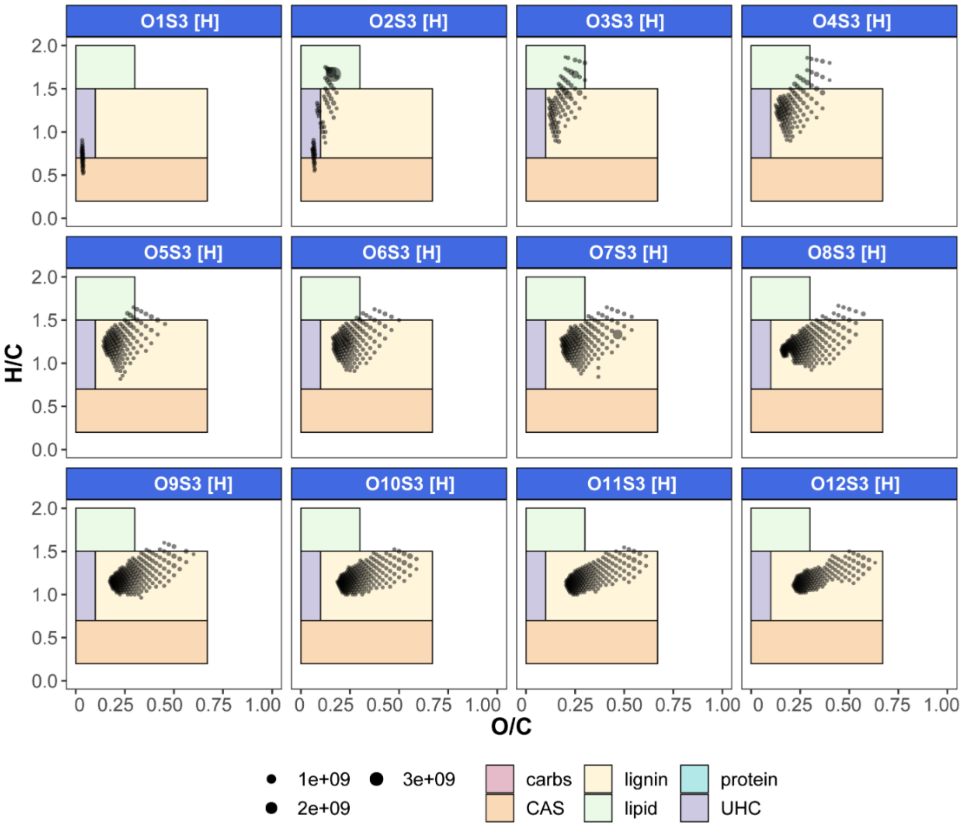

Fig. S35 Van Krevelen diagrams of O_OS_3[H] species detected in the unique in Bio-oil DMSO-Ac_2O.
